# “The measures taken by the government overburdened the daily practice” – insights of the PRICOV-19 study on German general practitioners in times of COVID-19

**DOI:** 10.1186/s12875-023-02115-4

**Published:** 2023-10-11

**Authors:** Stefanie Stark, Emmily Schaubroeck, Marie Kluge, Larissa Burggraf, Marco Roos, Eve Borowski, Esther Van Poel, Sara Willems, Thomas Kühlein, Susann Hueber, Felix Werner

**Affiliations:** 1 Institute of General Practice, Friedrich-Alexander University Erlangen-Nürnberg (FAU), Erlangen, Germany; 2 Department of Public Health and Primary Care, Ghent University, Ghent, Belgium; 3 Department for Sociology, University of Education, Schwäbisch Gmünd, Germany; 4 General Practice, Faculty of Medicine, University of Augsburg, Augsburg, Germany

**Keywords:** Primary health care, General practitioners, COVID-19, PRICOV-19, Quality of care, Health policy, Wellbeing

## Abstract

**Background:**

The international study PRICOV-19 aims to assess the impact of the COVID-19 pandemic on the organisation of primary health care. The German part focuses on German general practitioners during the second wave of the COVID-19 pandemic. This paper addresses the following research questions: (1) How were changes in tasks on primary care and patient treatment perceived by GPs?, (2) What was the role of GPs during the pandemic, and how was their wellbeing?, (3) How did GPs perceive health policy measures?, and, (4) What influenced the attitudes of GPs on health policy measures?

**Methods:**

This study pursues a multi-country cross-sectional design. Data collection took place throughout Germany from 01.02. to 28.02.2021 with a quantitative online questionnaire consisting of 53 items. The questionnaire was analysed through descriptive and inferential analyses using correlation and multiple regression models.

**Results:**

The response rate was 20.4% (*n* = 349). The respondents were mainly GPs (59.6%) in single practices (62.5%) with a mean work experience of 15 to 20 years. GPs experienced a change in their work and practice organisation (80.3%). They felt a high responsibility (70.6%) and found their work has become more meaningful to them (76%). They also saw a lack of political support (75.2%) and that the measures taken by the government overburdened the daily practice (66.4%). Not many GPs were at risk of being distressed (53.4%) but rated the health policies rather negatively (60%). The multiple regression showed, the more GPs were exposed to risk of distress, the worse they assessed the government's measures.

**Conclusion:**

GPs perceived their work as relevant and felt confident they could fulfil their tasks, but noticed that health policy initially hardly supported the outpatient sector. Health policies should increase their competence in relation to primary care, ensure its needs and consider an active inclusion of GPs in preparedness plans.

**Supplementary Information:**

The online version contains supplementary material available at 10.1186/s12875-023-02115-4.

## Introduction

Due to the rapid spread and unforeseen course of the pandemic, the high contagiousness of the SARS-CoV-2 virus, and the high mortality rate, the COVID-19 pandemic presented itself as a worldwide crisis [1–5]. Health systems were heavily exposed to this pandemic and faced new challenges to medical-professional interactions [5, 6]. In Germany, this crisis revealed who or what was coined "system-relevant" [7, 8], i.e., necessary for the maintenance and continued existence of the healthcare system [6]. The first COVID-19 wave (March 2020 – May 2020) hit the German health system mostly unprepared [9]. The health policy, as well as the system, was not sufficiently prepared at that time. The general public first noticed the effects of the pandemic in the public health sector, however the first place where the effects became visible was the clinical sector, especially the intensive care units (ICU). Initially, research, media and particularly political focus were primarily on this sector in its fight against the virus [10–14]. Nevertheless, a comprehensive approach to managing the COVID-19 pandemic should also focus on primary care, as the outpatient sector was also caught unprepared by this pandemic: no or inadequate personal protective equipment (PPE), no instructions or measures for pandemic preparedness (PP), and initially hardly or insufficient support from health policies [15]. Albeit at this time, six out of seven COVID-19 patients in Germany were mainly treated in primary care practices first [2, 14, 16].

Accordingly, the prolonged COVID-19 crisis has triggered government and policy reactions that intended to protect the ICUs from being overburdened by maintaining the primary care sector functioning [17, 18]. Especially with the beginning of the second pandemic wave (September 2020 – February 2021) in Germany [9], primary care played an essential role in the care of infected patients and was put at the forefront of providing essential support in containing and diagnosing COVID-19 [19–21]. New regulations posed organisational, structural, and content-related challenges in German general practices [21–23]: e.g., the emergency service being used for COVID-19 testing, as well as the implementation of special infection consultation hours [24]. Primary care was set to be the first point of contact where decisions had to be taken on whether patients should be referred to the hospital for further treatment. Patients with less severe and uncomplicated symptoms were entirely cared for in primary care [25, 26]. Furthermore, preventive care, e.g., vaccinations, was widely accomplished by General practitioners (GPs) in their practices [19, 20, 27, 28]. In this pandemic, the primary care sector had to maintain basic health care structure, especially if other parts of the healthcare system were in danger of overloading [4, 21, 29]. Therefore, German GPs functioned as gatekeepers who provided authorisation for access to hospital care by mitigating the risk of overburdened clinics and delayed specialist care [13, 30, 31]. Former studies have shown that primary care and its actors were under intense pressure and faced new challenges and difficulties during the COVID-19 pandemic [23, 26, 32] and has thus presented new and fast-changing tasks for GPs to maintain outpatient care [19, 21, 26, 33]. Furthermore, studies regarding previous pandemics demonstrated a negative impact on the GPs’ perception of their role. Accordingly, previous research also showed that the COVID-19 pandemic, with its policy measures as well as its regulations, negatively affected the GPs’ workload and, subsequently, their wellbeing [34, 35].

The international collaboration study *PRICOV-19 – Primary Health Care in times of COVID-19*^1^ aims to assess the impact of the COVID-19 pandemic on the organisation of primary health care. The German part of this study focuses on the impact of the pandemic measures and health policy regulations on GPs’ perceptions and wellbeing during the second wave of the COVID-19 pandemic in Germany (February 2021). It is assumed from the research aforementioned that pandemic-related changes due to associated political actions would manifest themselves in the role and tasks of the GPs, affecting their workload, as well as their mental wellbeing. Accordingly, this paper addresses the following research questions: (1) *How were changes in tasks on primary care and patient treatment perceived by GPs?*, (2) *What was the role of GPs during the second wave of the pandemic, and how was their wellbeing?*, (3) *How did GPs perceive the health policy measures?*, and, (4) *What influenced the perceptions and attitudes of GPs on health policy measures?*

## Methods

### Study design and setting

The PRICOV-19 study uses an online self-reported survey to conduct a multi-country cross-sectional design in 38 European countries [36]. In Germany, this study was conducted by the Institute of General Practice of the Friedrich-Alexander University Erlangen-Nürnberg (FAU).

### Development, translation and validation of the questionnaire

The overall online questionnaire for all participating countries of this study was developed at Ghent University in multiple phases, including a pilot study among 159 GP practices in Flanders (Belgium). This questionnaire was developed based on a literature review and theoretical framework on quality of care [37] and patient safety culture [38]. More details are described in the study protocol (Additional File 1), including the questionnaire as an additional file [36]. The validated English questionnaire was provided to each research partner to be translated into the country’s primary language. In Germany, we used a forward-backwards method to guarantee contextual conformity in translating the questionnaire into German. Two German GPs from the Institute of General Practice Erlangen independently reviewed the translated questionnaire and adapted it where necessary. Furthermore, each country could add up to ten items to the questionnaire to pursue its own research question and focus on the context of its own country, as well as primary care and health system. The amended German questions were tested for comprehensibility through cognitive interviews [39] with four GPs and two non-GPs (research associates).

### Structure of the questionnaire and measures

The main questionnaire (Additional Files 2 and 3) consisted of 53 items comprising seven topics, as seen in Table 1. Participants could also add further comments, suggestions, and feedback in an open text field. The open text field was the last item of the questionnaire, its limit was 500 words. All participants gave written informed consent on the first page of the online survey.

**Table 1:** Main content areas and items of the questionnaire in the respective order^a^

Content	Sample item
1.0 General information about the respondent and primary care	What is your position in this GP practice?
1.1 Patient flow during and before the COVID-19 pandemic:	
a. Appointments	a. Patients who made an appointment and where it is unclear whether they pose a risk of infection are called to verify this
b. Triage	b. In case the telephonic triage is performed by someone other than a GP in this GP practice and he/she needs support when assessing a call, he/she can rely on support from a GP
c. Safety management for routine primary care	c. A patient with a fever caused by an infection other than COVID-19 was seen late due to the COVID-19 protocol
1.2 Infection prevention	In this GP practice, home care nurses are currently actively contacted when their patients are diagnosed with a major infectious disease (e.g. HIV, COVID-19, hepatitis carrier status)
1.3 Information processing	In this GP practice, a fixed weekly time is provided in the agenda(s) of GPs for reviewing new guidelines or going through relevant and reliable scientific literature
1.4 Communication with patients	Does this GP practice have a brochure with information on COVID-19 to give to patients?
1.5 Dealing with health policy measure	The guidelines imposed by the government on GP practices as a consequence of COVID-19 pose a threat to the good organisation of this practice
1.6 Wellbeing	The guidelines imposed by the government on GP practices as a consequence of COVID-19 pose a threat to the personal wellbeing of the staff in this practice

^a^All questions were answered with a 5-point or 7-point Likert scale using an agreement scale or could be answered with yes/no

Furthermore, another three topics with ten items were added to the German version of the questionnaire (Additional Files 4 and 5), which mainly dealt with the context of primary care in Germany (Table 2). The focus of these particular questions lies primarily on the impact of the German health policy measures on primary care from the perspective of the GPs.

**Table 2:** Items added for Germany in particular^a^

Content	Sample item
2.0 Implementation feasibility of standard care during COVID-19	The care of COVID (suspected) cases cannot sufficiently ensure the care of uncomplicated diseases (e.g. back pain, urinary tract infection)
2.1 Acceptance of political measures/interference of politics and society in the GP profession	The measures taken by the government concerning GP care to contain the pandemic have overwhelmed everyday practice The role of GPs has gained attention in society since the beginning of the pandemic
2.2 Structural changes in GP practice due to policy measures	The local structures of medical cooperation (e.g. interprofessional exchange, substitute organisation) have changed positively as a result of the pandemic

^a^All questions were answered with a 5-point Likert scale using an agreement scale

### Recruitment and sample

The recruitment in Germany occurred nationwide from 01.02.2021 to 28.02.2021. The target (n) for Germany was 200 practices, following Ghent University requirements on specific ratios per country (*n* = 200 for countries with more than 10,000 GPs). We assumed a response rate of 10%. The online questionnaire was directed to GPs, internists working in primary care, and GP trainees who work in single or group practices in rural, suburban or urban settings. For better readability we will refer in the following to all participants as GPs. We recruited in a snowball procedure to gain quick and broad access to the research field via anonymised e-mail dispatch lists from the general practice institutes’ research practices (FAU Erlangen), other general practice institutes (throughout Germany), regional German general practices networks, as well as the Bavarian general practitioners’ association. The participants could access the questionnaire through a link, which guided them to the online platform REDCap [40]. No incentive was given to the participants, and no reminders were sent. We sent out the survey link to 1,710 GPs. To track the data collection and the targeted n, weekly updates were sent from the REDCap server regarding the number of the already completed questionnaires. All data were collected anonymously and stored on the secure Ghent University data servers [41].

The reporting of this study is based on the *STROBE* (Strengthening the Reporting of Observational Studies in Epidemiology) recommendations [42] and the *CHERRIES* (Checklist for Reporting Results of Internet E-Surveys) checklist [43].

### Data analysis

All statistical analyses were carried out with IBM SPSS Statistics 28. Boundary values of *p* < 0.05 were set for statistical significance. Data and syntax can be found in the supporting information files (Additional Files 6 and 7).

Firstly, descriptive analyses were conducted for the following topics: sample and cohort characteristics, perceiving the changes in tasks on primary care and patient treatment, perceiving the role of GPs and task shifting, perceiving the wellbeing of GPs (expanded 9-item WellbeingIndex, eWBI), as well as the assessment of the health policy measures (PolicyIndex, PI).

Secondly, two additive indices were created: The eWBI describes the frequency of distress and wellbeing among healthcare professionals. In this paper, the validated expanded 9-item Wellbeing Index version was used according to Dyrbye [44]. Cronbach's α was used to check whether all variables could be combined into an additive index (Table 6, Items: 6.0—6.8). The internal consistency was satisfying, with *Cronbach's α* for a positive effect of 0.76 for the eWBI. Within the eWBI, seven items had to be responded to with a yes (scored as 1) or no (scored as 0). Two items that had to be answered on a 7-point Likert scale were converted into a new variable: response options of a scale of 1 or 2 (a low level of meaning in work) were assigned the value + 1, response options of 3 to 5 (a neutral level of meaning) was given 0, and response option of 6 or 7 (a high level of meaning in work) was assigned the value -1. The 5-point Likert scale was recoded to the same scheme: those responding ‘strongly disagree/disagree’ having + 1, those who responded ‘agree/strongly agree’ to having -1, and 0 for those with middle neutral responses. As in previous studies being at risk of distress is defined as a score of ≥ 2 [44]. The PI was built on the same basis as the eWBI. The PI was used to aggregate the GPs' perceptions and attitudes towards health policy measures on primary care during the pandemic. It was composed of the variables of a 5-point Likert scale (Table 8, Items: 8.0—8.3). The internal consistency was proved satisfying, with *Cronbach's α* for a positive effect of 0.69.

Thirdly, inferential analyses were conducted using correlation and multiple regression models. *Bravais-Pearson* [45] correlation was first calculated to see if the two indices correlate to ensure they can be used in the regression model. Regarding the multiple regression model analysis, we used the ones with the best model quality (*R*^*2*^). Three models were calculated, with the metric *PI* as the dependent variable. The independent variables were introduced in three steps: (1) variables that deal with the change of role and tasks of GPs (2), the eWBI, and (3) the practice-relevant influencing factors (cohort characteristics).

## Results

### Sample and cohort characteristics

Three hundred forty-nine GPs filled in the questionnaire (Item 3.0) with an estimated response rate of 20.4%. At least, we had 250 fully completed questionnaires. Participants who filled out the questionnaire were mainly GPs (59.6%, Item 3.1) with mean work experience of 15 to 20 years (Item 3.2). The majority worked in single practices (62.5%, Item 3.3). Most practices were located in rural areas or in small towns (53.3%, Item 3.4). For further details please see Table 3.

**Table 3:** German cohort characteristics

	**N**	**%**
3.0 Participants	349	100
3.1 Position/Function		
GP	208	**59.6**
Internists as GP	43	12.3
GP trainees	8	2.3
Missing	90	25.8
3.2 Work experience in years		
< 5	30	8.6
5—< 10	26	7.4
10—< 15	33	9.5
15—< 20	44	**12.6**
20—< 25	48	13.8
25—< 30	34	9.7
30—< 35	23	6.6
> 35	12	3.4
Missing	99	28.4
3.3 Practice type		
Single	218	**62.5**
Group	111	31.8
Missing	20	5.7
3.4 Practice location		
Rural	91	**26.1**
Small Town	95	**27.2**
Urban	76	21.8
Missing	87	24.9

### Perceived changes in tasks on primary care and patient treatment

 GPs agreed that priority was given to suspected COVID-19 cases over other patients in terms of appointments (63.3%, Item 4.0). Also, a high proportion of respondents indicated that patient care for uncomplicated cases/diseases was ensured (85%, Item 4.1). GPs experienced that the impact of the pandemic increased recommending non-COVID-19 vaccinations, e.g. influenza or pneumococcus (70.7%, Item 4.8), and requests for COVID-19 testing by asymptomatic patients (62.2%, Item 4.9). For further results, please see Table 4.

**Table 4:** Perceived changes in tasks on primary care and patient treatment^a^

	***n***	**Strongly disagree**	**Disagree**	**Neutral**	**Agree**	**Strongly Agree**	**Mean (SD)**
4.0 COVID-19 suspected cases are given priority when appointments are made.	256	6 (2.3%)	31 (12.1%)	57 (22.3%)	**76 (29.7%)**	**86 (33.6%)**	3.8 (1.1)
4.1 Care for suspected COVID-19 cases does *not* adequately ensure care for other uncomplicated cases/diseases (e.g. back pain).	259	**89 (34.4%)**	**131 (50.6%)**	22 (8.5%)	14 (5.4%)	3 (1.2%)	1.9 (0.85)
4.2 Patients with uncomplicated diseases are mainly treated by telephone consultations (e.g. back pain).	260	**76 (29.2%)**	**127 (48.8%)**	32 (12.3%)	21 (8.1%)	4 (1.5%)	2.0 (0.94)
4.3 Suspected Covid-19 cases were mainly treated via telephone consultations.	259	**69 (26.6%)**	**81 (31.3%)**	28 (10.8%)	57 (22.0%)	24 (9.3%)	2.6 (1.34)
4.4 *No* appointments are offered for routine consultations/examinations (e.g. back pain).	259	**116 (44.8%)**	**103 (39.8%)**	14 (5.4%)	18 (6.9%)	8 (3.1%)	1.8 (1.02)
4.5 The possibility of a telephone or video consultation relieves the burden on practice resources.	254	29 (11.4%)	68 (26.8%)	52 (20.5%)	**77 (30.3%)**	**28 (11.0%)**	3.0 (1.21)
4.6 Since the beginning of the pandemic, the practice has increasingly offered home visits to patients at risk.	259	**36 (13.9%)**	**110 (42.5%)**	48 (18.5%)	58 (22.4%)	7 (2.7%)	2.6 (1.07)
4.7 The cooperation between local practices have changed positively as a result of the pandemic (e.g. professional exchange, support).	258	**47 (18.2%)**	**105 (40.7%)**	67 (26.0%)	31 (12.0%)	8 (3.1%)	2.4 (1.02)
4.8 Protective vaccinations are increasingly recommended by the practice (e.g. influenza, pneumococcus).	259	5 (1.9%)	17 (6.6%)	54 (20.8%)	**115 (44.4%)**	**68 (26.3%)**	3.9 (0.95)
4.9 The request for COVID-19 testing *by* asymptomatic patients is increasing.	258	3 (1.2%)	36 (14.0%)	36 (14.0%)	**103 (39.9%)**	**80 (31.0%)**	3.9 (0.94)
4.10 COVID-19 testing *in* asymptomatic patients is more increasing as the pandemic progresses.	257	6 (2.3%)	49 (19.1%)	42 (16.1%)	**100 (38.9%)**	**60 (23.3%)**	3.6 (1.12)

^a^5-point Likert scale

### Perceived role of GPs and task shifting

Almost 40% of the GPs agreed that their role had gained more attention. However, an equal proportion also said that they did not perceive this to be the case (Item 5.0). 76% of the respondents perceived their work during the pandemic had a personal added value for them and became more meaningful to them (Item 5.1). Next, 80.3% of them reported that their role and tasks in primary care had changed since the beginning of the pandemic (Item 5.2), with increased responsibilities (70.6%, Item 5.3). Not even half of them felt prepared for the task shifting in their professional role (49,2%, Item 5.5). 42.2% stated that they were unhappy with the task shifting in their professional role (Item 5.4). Further results can be seen in Table 5.

**Table 5:** Perceived role of GPs and task shifting^a^

	***n***	**Strongly disagree**	**Disagree**	**Neutral**	**Agree**	**Strongly Agree**	**Mean (SD)**
5.0 The *role of GPs* has *gained attention* since the beginning of the COVID-19 pandemic.^a^	255	**22 (8.6%)**	**71 (27.8%)**	61 (23.9%)	**81 (31.8%)**	**20 (7.8%)**	3.0 (1.12)
5.1 Since the COVID-19 pandemic, *the work* I do has *become more meaningful* to me.^a^	258	5 (1.9%)	17 (6.6%)	40 (15.5%)	**107 (41.5%)**	**89 (34.5%)**	4.0 (0.97)
5.2 *Primary care has changed* since the COVID-19 pandemic (e.g. more medical attestations, communication with health authorities).^a^	259	3 (1.2%)	17 (6.6%)	31 (12.0%)	**117 (45.2%)**	**91 (35.1%)**	4.1 (0.92)
5.3 Since the COVID-19 pandemic, my *responsibilities* in this practice *increased*.^a^	248	2 (0.8%)	17 (6.9%)	59 (23.8%)	**87 (35.1%)**	**83 (35.5%)**	2.9 (0.96)
5.4 I am *happy with the task shifting in my professional role* since the COVID-19 pandemic	242	**18 (7.4%)**	**80 (34.8%)**	66 (21.4%)	64 (30.6%)	14 (5.8%)	2.0 (1.04)
5.5 I *do not feel prepared for the task shifting in my professional role* since the COVID-19 pandemic.^a^	246	**50 (20.3%)**	**71 (28.9%)**	49 (18.0%)	45 (24.2%)	31 (8.5%)	1.6 (1.2)

^a^5-point Likert scale

### Perceived wellbeing of GPs (expanded 9-item WellbeingIndex, eWBI)

Half (50.5%) of the GPs stated that they felt burned out from their work (Item 6.0) and had less time for leisure time activities (38.0%, Item 6.8). 88.5% said their work during the pandemic was meaningful to them (Item 6.7). More details please see Table 6.

**Table 6:** Perceived wellbeing of GPs (*eWBI components*)^a^

	***n***	**%**
6.0 Have you felt burned out from your work?		
No	121	49.6
Yes	123	**50.4**
6.1 Have you worried that your work is hardening you emotionally?		
No	149	**61.3**
Yes	94	38.7
6.2 Have you often been bothered by feeling down, depressed, or hopeless?		
No	169	**69.3**
Yes	75	30.7
6.3 Have you fallen asleep while sitting inactive in a public place?		
No	231	**95.5**
Yes	11	4.5
6.4 Have you felt that all the things you had to do were piling up so high that you could not overcome them?		
No	184	**76.0**
Yes	58	24.0
6.5 Have you been bothered by emotional problems (such as feeling anxious, depressed, or irritable)?		
No	145	**59.4**
Yes	99	40.6
6.6 Has your physical health interfered with your ability to do your daily work at home and/or away from home?		
No	200	**83.0**
Yes	41	17.0
6.7 The work I do is meaningful to me.^a^		
1 (Strongly disagree)	-	-
2	-	-
3	2	0.8
4	7	2.9
5	19	7.8
6	80	32.9
7 (Strongly agree)	135	**55.6**
6.8 My work schedule leaves me enough time for my personal/family life.^b^		
1 (Strongly disagree)	26	10.7
2	66	**27.3**
3	59	24.4
4	63	**26.0**
5 (Strongly agree)	28	11.6

^a^7-point Likert scale

^b^5-point Likert scale

The risk of distress is defined with a score of ≥ 1 (*median*) according *to *Dyrbye [44]. Considering the eWBI, the mean is at + 1.2 (*SD* = 2.6), with 53.4% of the GPs situated above + 1.0. This indicates that more than half of our GPs surveyed were at risk of distress due to COVID-19 during the second wave of the pandemic in Germany.

### Perceived health policy measures (PolicyIndex, PI)

Around 44.5% of the respondents agreed that the government's policies and regulations on primary care practices threatened the good practice organisation (Item 7.0). Besides that, 43.3% reported that the policies threatened the personal wellbeing (Item 7.1). Furthermore, the majority agreed that no adequate government support was given (75.2%, Item 7.2), and 66.4% considered that the policies overwhelmed and negatively affected the daily life of their practice (Item 7.2). Please see Table 7.

**Table 7:** Perceived health policy measures (*PI components*)^a^

	***n***	**Strongly disagree**	**Disagree**	**Neutral**	**Agree**	**Strongly Agree**	**Mean (SD)**
7.0 The guidelines imposed by the government on primary care practices as a consequence of COVID-19 *pose a threat to the good organisation* of this practice.^a^	246	30 (12.2%)	66 (26.8%)	55 (16.4%)	**79 (38.0%)**	**16 (6.5%)**	1.9 (1.1)
7.1 The guidelines imposed by the government on primary care practices as a consequence of COVID-19 *pose a threat to the personal wellbeing.*^a^	247	52 (21.1%)	24 (9.7%)	64 (25.9%)	**37 (15.0%)**	**70 (28.3%)**	1.8 (1.2)
7.2 *Adequate support is provided* by the government for the proper functioning of this practice.^a^	246	**90 (36.6%)**	**95 (38.6%)**	40 (16.3%)	14 (5.7%)	7 (2.8%)	1.0 (1.0)
7.3 The measures taken by the government have *overburdened daily practice.*^a^	254	15 (5.9%)	56 (22.0%)	55 (5.6%)	**84 (39.1%)**	**44 (27.3%)**	3.3 (1.2)

^a^5-point Likert scale

The PI index has a range from -4.00 to + 4.00 points. The lower this score, the more GPs agreed with the health policy measures; the higher this score, the less GPs agreed with the government’s policies and were more dissatisfied. The *mean* was + 0.7, and the *median* was + 1.0. We defined being less in agreement with the policy measures as a score of ≥ 1 (*median*) according to Dyrbye [44]. Almost 60% of GPs were above the cut-off point of + 1.0, meaning that there was a rather high degree of dissatisfaction among the German GPs with the health policies during the second wave of the COVID-19 pandemic.

### Correlation of eWBI and PI

Table 8 shows a highly significant two-sided relationship between the eWBi and PI indices. *R* = 0.44 corresponds to a medium negative effect, e.g., the higher the eWBI score (and the lower the wellbeing), the more negative the GP’s health policy attitude. In the context of our previous results, this means that in the second COVID-19 wave in Germany, we found that when GPs were in greater distress, they rated the health policies more negatively.

**Table 8:** Bravais-Pearson-Correlation between eWBI and PI

	**PolicyIndex**
**WellbeingIndex**	
Pearson-Correlation *r*	0.441^a^
Sig. (two-sided)	**< 0.001**
n	225

^a^The Correlation is significant at 0.01 (two-sided)

### Multiple linear regression models

All three models (Table 9) were highly significant (*p* < 0.001). In the first model, a significant influence on GPs' health policy attitudes was exerted above all by the perception of whether one felt happy with the task shifting (*β* = -0.249, *p* < 0.001) and whether one felt unprepared (*β* = 0.223, *p* < 0.001). The most substantial negative influence was dissatisfaction with the change of tasks in practice, connecting a lower satisfaction to a higher PI score. In the second model, adding the factor eWBI, which had a highly significant influence on the PI (*β* = 0.347, *p* < 0.001), reduced the impact of the previously significant variables. This means that the more GPs were exposed to the risk of distress during the pandemic, the worse they assessed the government's measures. In the third model, the significance of the eWBI (*β* = 0.352, *p* < 0.001) did not change by adding the cohort characteristics, which did not influence the PI. We therefore assume that the cohort characteristics have no influence on the PI, neither directly nor indirectly mediating via the eWBI. It can be assumed that the GPs assessed the health policy measures irrespective of their function, practice location, type of practice, or work experience. The respondents' wellbeing predominated as the most influential variable.

**Table 9:** Results of linear mixed model analysis of potential predictors for the GP’s perception of health policy measures (PI score, PolicyIndex) during the pandemic

**Linear Mixed Models for Total PI score (***PolicyIndex***)**					
**A. Model summary**					
	**CorrelationCoefficientr**	**r**^**2**^	**Adjusted r**^**2**^***p***		
**Modell 1**	0.395	0.156	0.139 < **0.001**		
**Modell 2**	0.505	0.255	0.237 < **0.001**		
**Modell 3**	0.515	0.265	0.233 < **0.001**		
**B. Predictor coefficients**					
	**Regression Coefficient *B***	**Standardised error**	***β***	***t***	***p***
***Modell 1***					
(Constant)	0.193	0.767	-	0.252	0.802
Primary care changed^a^	1.203	0.633	0.127	1.900	0.059
Responsibilities increased^a^	-0.267	0.636	-0.028	-0.419	0.675
Happy with task shifting^a^	-1.197	0.316	-0.249	-3.787	**< 0.001**
Not feeling prepared^a^	1.015	0.299	0.223	3.394	**< 0.001**
***Modell 2***					
(Constant)	0.315	0.723	-	0.436	0.663
Primary care changed^a^	0.914	0.599	0.097	1.527	0.128
Responsibilities increased^a^	-0.535	0.601	-0.057	-0.890	0.375
Happy with task shifting^a^	-0.821	0.306	-0.170	-2.680	**0.008**
Not feeling prepared^a^	0.578	0.294	0.127	1.966	**0.050**
WellbeingIndex (eWBI)	0.312	0.060	0.347	5.222	**< 0.001**
***Modell 3***					
(Constant)	0.908	0.898	-	1.011	0.313
Primary care changed^a^	0.936	0.604	0.099	-0.868	0.123
Responsibilities increased^a^	-0.527	0.607	-0.056	-2.794	0.387
Happy with task shifting^a^	-0.863	0.309	-0.179	1.829	**0.006**
Not feeling prepared^a^	0.543	0.297	0.119	5.262	0.069
WellbeingIndex (eWBI)	0.317	0.060	0.352	5.262	**< 0.001**
Function: GP^b^	-0.341	0.369	-0.058	-0.925	0.356
Practice location: rural^b^	0.193	0.278	0.042	0.693	0.489
Type of practice: single^b^	-0.499	0.394	-0.078	-1.268	0.206
Work experience: less than 15 years^b^	0.072	0.295	0.015	0.245	0.807

^a^The following 5-point Likert scale items were converted into dummies: 6.3 *Primary care has changed since the COVID-19 pandemic. 6.4 Since the COVID-19 pandemic, my responsibilities in this practice increased. 6.5 I am happy with the task shifting in my professional role since the COVID-19 pandemic. 6.6 I do not feel prepared for the task shifting in my professional role since the COVID-19 pandemic*

^b^The following items were converted into dummies: *4.2 Position/Function. 4.3 Work experience in years. 4.4 Practice type. 4.5 practice location*

## Discussion

Our main findings show that the German GPs experienced a considerable change in their work and practice organisation, as well as rapid changes in tasks. They felt a high responsibility within the COVID-19 pandemic and found that their work has become more meaningful since the pandemic. On contrary the GPs also saw a lack of political support, and many felt that they were not well prepared by the government. Furthermore, they found that the measures taken by the government have overburdened the daily practice. However, the wellbeing index showed that many respondents did not feel very strongly affected by their psychological wellbeing, but rated the implementation of the policies and especially the impact on the organisation of the practice rather negatively. The GPs' wellbeing is significantly related to how the respondents rated the German health policies: The more dissatisfied the GPs were with the task shifting, the more negative they felt about the policies decided from the government. It follows, that from the perspective of the German GPs, the health policy measures had a negative influence on everyday practice rather than being supportive. If the practice team and the GPs felt unprepared, experienced little support by the government and were dissatisfied with the changes in tasks, they evaluated the political actions more negatively. It could be deduced that if GPs were dissatisfied and overwhelmed with the measures, they may be less able to implement them. Accordingly, active inclusion of the GPs in health policy decisions and recommendations for action and a participatory approach should be considered.

A study by Wangler et al*. *[46] showed that GPs, based on their own insights and experiences, do not just want advice, action and suggestions from other experts, such as policymakers. Therefore, it seems advisable to involve GPs and their experience in the planning, implementation and evaluation of measures to preparedness planning and in contingency plans [46]. Other studies [15, 21] have also highlighted the need to involve GPs in preparedness planning and showed that GPs gave low ratings to their preparedness for a pandemic. According to Siebenhofer et al. (2021), primary care is important for dealing with pandemics like COVID-19. GPs in that study said that they were confident and willing to take an active role, but they need to be provided with the appropriate ambient conditions, and information and especially be included in communicative exchange [21]. Other research [10, 47] also demonstrated that the COVID-19 pandemic had negatively affected healthcare workers in the primary ambulatory sector. Those seemed to be the most vulnerable group due to increasing responsibilities and transforming tasks. Similar findings [34, 35, 48] showed that GPs experienced a new role marked by a high-stress level combined with a deleterious work environment caused by the COVID-19 pandemic and its health policy measures. Collinset al*.* [34]*,* concluded within the international comparison of the PRICOV-19 study, that GPs with less experience, in smaller practices, and with more vulnerable patient populations had higher scores in the eWBI, thus being at a higher risk of distress. Furthermore, Groenewegen et al. (2022) found, that all over Europe, GPs and their staff members were more involved in giving information and recommendations to patients contacting the practice by phone, they were more engaged in triage of whom to refer to the hospital, and that they experienced more responsibilities [26].

### Strengths and limitations

The strengths of our study are that it represents a gap in research on German primary care during the second wave of the COVID-19 pandemic in February 2021. It gives an important snapshot of the primary care practices within this time frame with a validated questionnaire and a high response rate. Furthermore, regarding our data and as it is an international cross-sectional study design, comparison with other countries becomes possible and could be an incentive for further evaluation and analysis steps.

We also see several limitations to this work. It can be assumed that the GPs who took part in the survey were rather more dissatisfied with the situation than the basic population. We therefore assume the existence of a selection bias. However, the demographic data collected does not indicate a clear major shift in the composition of the sample and the basic population. Additionally, we expect further biases, e.g. social desirability and dependence on the familiarity of the respondent with the practice organisation. Moreover, within our study, no comparison can be made between the situation before and after the pandemic and its waves.

## Conclusion

The COVID-19 pandemic led to new challenges, barriers and responsibilities for German primary care. During the second wave of the pandemic, the German GPs of this study perceived their work as relevant and felt confident that they could fulfil their tasks. Nevertheless, they noticed that health policy initially hardly reacted or was delayed in reacting and supporting the outpatient sector. This led to an increased risk of distress and dissatisfaction on the part of the GPs, as an overload and high workload became apparent in the primary care setting as well, and not only at ICU level. High-quality outpatient care during the pandemic could initially only be maintained through improvisation and extreme flexibility on the part of the GPs. What was needed was a transparent pandemic plan, including the primary care sector. Consequently, it is recommended that health policy and its actors ought to take an active interest in understanding the complexity of GPs' work. They could increase their competence in relation to the primary care service, ensure that the needs of primary care are considered in preparedness plans (pandemics, climate change etc.), and include the GPs expertise concerning health policy questions.

### Supplementary Information

**Additional file 1.** Study protocol.

**Additional file 2.** Main questionnaire, English.

**Additional file 3.** Main questionnaire, German.

**Additional file 4.** Items added, English.

**Additional file 5.** Items added, German.

**Additional file 6.** Data.

**Additional file 7.** Syntax.

## Data Availability

All relevant data is available at BMC online and within this paper as Supporting Information files.

## Abbreviations

ICU: Intensive care units
PPE: Personal protective equipment
PP: Pandemic preparedness
GPs: General practitioners
PRICOV-19: Primary health care in times of COVID-19
STROBE: Strengthening the Reporting of Observational Studies in Epidemiology
CHERRIES: Checklist for Reporting Results of Internet E-Surveys
eWBI: Expanded 9-item WellbeingIndex
PI: PolicyIndex

## Footnotes

1 The PRICOV-19 study is a multi-country cross-sectional study researching how primary care practices were organised during the COVID-19 pandemic to guarantee safe, effective, patient-centred, and equitable care; and the pandemic's impact on the role and wellbeing of care providers. These aspects were examined through an online questionnaire.
