# Supplementary material for: “The measures taken by the government overburdened the daily practice” – insights of the PRICOV-19 study on German general practitioners in times of COVID-19
Source: BMC Prim Care. 2023 Oct 11;24(Suppl 1):207. doi: 10.1186/s12875-023-02115-4 (PMC10568746; doi:10.1186/s12875-023-02115-4)
Supplement: Supplementary file 2 — Additional file 2. Main questionnaire, English. [file 12875_2023_2115_MOESM2_ESM.pdf]

Quality of care and patient safety in GP practices in times of the Covid 19 pandemic

Dear Ladies and Gentlemen,

the COVID-19 pandemic has presented GPs with unforeseeable organisational and content-related challenges, including changed tasks such as telemedicine, regional cooperation with the ÖGD, contract physicians and inpatient facilities and limited availability of human and infrastructural resources as well as a lack of personal protective equipment (PPE). The changed field of work also poses a challenge for the quality of care in its dimensions: Patient safety, effectiveness, patient-centredness, timeliness, efficiency and health equity.

The PRICOV-19 study is being conducted in more than 36 countries to assess the impact of the COVID-19 pandemic on the organisation of care in GP practices. In addition, it will be investigated how access to patient care was ensured and what influence the pandemic had on the quality of care. This study is being carried out internationally with several cooperation partners from different countries; the study is being led by Ghent University.

Your participation in this study means a lot to us! The results will support policy makers across Europe on what measures will improve care in the outpatient sector in a pandemic.

Participation in this study is anonymous and your answers cannot be traced back to you. Your data will be kept by Ghent University and will only be shared with the cooperating researchers. Of course, your data will be handled according to the European Data Protection Regulation, will not be shared with third parties and will be destroyed after 20 years.

All research results are made publicly available via the websites of the participating research groups, through scientific publications and other communication channels.

The questionnaire will take about 20 minutes to complete. We thank you in
advance for your support!

Yours sincerely,

Prof. Dr Thomas Kühlein
Stefanie Stark General Practice
Institute
Friedrich Alexander University Erlangen-Nuremberg

Prof. Dr Sara Willems
Principal Investigator
Department of Public Health and Primary Care, Ghent University, Belgium

Completion aid

Please note the following instructions for completing the questionnaire:

- Do you work in different practices or at several locations? Then fill in the questionnaire with reference to a specific practice and location.
- The term 'staff' in the questionnaire refers to all those who actively work in this practice. This includes both paid and unpaid staff.
- The questionnaire should preferably be completed by a GP or a doctor in training. You can also complete the questionnaire as a team in a team meeting. In this case, the questionnaire can even act as a trigger for team-internal discussions about quality assurance in times of COVID-19.
- There are no wrong answers. The answer options may not contain the answer that exactly reflects your situation in practice. In this case, please choose the answer option that most closely corresponds to the current situation in your practice.
- The questionnaire consists of six parts: Background information, patient pathways, infection prevention, information processing, patient information, and lastly cooperation, collegiality and self-protection.
- Only one questionnaire per practice should be completed.

It takes about 20 minutes to complete the questionnaire.

Declaration of consent

I have read and understood the information I received about this study.

- Yes
- No

I agree to participate in this study:

- Yes
- No

Part 1: Background information

The following questions relate to you and the structures of this practice. The questions that follow serve to assess the current situation in the GP practice (hereafter referred to as the 'this practice').

1. What is your position in this practice?
   - Specialist in general medicine, general practitioner
   - Specialist in internal medicine (family doctor/internist)
   - Doctor in training
   - This questionnaire is completed as a practice team
   - Other

Explain 'Other': ________________________

2. How many years of work experience do you already have in the field of general practice after completing your training as a specialist in general medicine (*)?

- Year(s):________________(Write a number).
- Month(s):________________(Write a number).

(*) If you have worked in the GP sector for 1 year and 2 months, please fill in 1 year(s) and 2 month(s).
(This item is only given by doctors, doctors in further training and 'other' as in question 1. filled)

3. How many people are actively working in this practice? (Including you and whether paid or unpaid).

- Number of paid employees (*) ____________________(Write a number).
- Number of unpaid employees (**):____________________(write a number).

(*) Paid employees: e.g. paid trainees, administrative staff, MFAs, General practitioners
(**) Unpaid staff: e.g. students, PJ-ler, interns

4. How many GPs and doctors in training work in this practice? Count each GP and doctor in training, regardless of their full-time equivalence (FTE). Do not forget to count yourself.

- Number of general practitioners/family doctors: ________(Write a number).
- Number of doctors in further training: _________________________ (Write a number).

5. How many full-time equivalent GPs (FTE) work in this practice(*)? Please indicate all GPs, including those in training, and do not forget to count yourself. Please indicate the number in decimal format (e.g. 2.5).

- Number of FTEs:________________(Enter a number).

(*) Please keep in mind that we are asking for FTEs. So if a GP works three days a week, that is an FTE of 0.6.

6. Which of the following professional groups are represented in this practice? (Don't be surprised, some of these are common in GP practices in other European countries).
(Indicate all occupational groups currently represented).

- Family doctor/family physician
- Doctor in training
- Medical assistant
- Practice manager
- Receptionist, Administrative Assistant
- Social worker
- Cleaning power
- Podiatrist
- Psychologist
- Nurse or auxiliary nurse
- Physiotherapist, manual therapist or osteopath
- Dietician or nutritionist
- Other

Explain 'Other': ________________________

7. What is the prevailing fee system(*) in this practice?

- Public Scale of Fees (GOÄ) Uniform
  Scale of Assessment (EBM)
- Selective contract (n. §73b SGB V)
- Other

Explain 'Other': ________________________

(*) Prevailing fee system: The one with the highest percentage of income

8. Are the GPs in this practice self-employed or in permanent employment? Please indicate all options that apply.

- paid employment
- Self-employed with a contract with a health insurance fund (in Germany with health insurance fund approval)
- Self-employed without contract (pure private practice)

(This item is only filled in by doctors as indicated in question 1)

9. How would you describe the situation of your practice?

- Big city (city centre)
- Suburb
- (Small) town
- Urban-rural mixed
- Rural

10. Have you perceived any constraints on the premises or infrastructure of this practice that have prevented you from providing safe and quality care since the onset of the COVID-19 pandemic?

- To a large extent
- In some parts
- Hardly
- None
- Not specified

11. With regard to the premises or infrastructure of this practice, has the COVID-19 pandemic led to any changes being considered in this regard?

- To a large extent
- In some parts
- Hardly
- None
- Not specified

12. We would like to get an idea of the size of this practice. Give an estimate of your total patient population (NOT number of notes per quarter).

- Number of patients: _______________(Write a number.)

13. Compared to the average GP practice, would you say that on average this practice sees more/less patients in the categories listed below?

- Above average
- About average
- Below the average
- I do not know

1. Patients with a migration background or those who have difficulty speaking the local language.
2. Patients with limited health literacy (*) or low literacy (**).
3. Patients with financial problems.
4. Patients with psychiatric problems.
5. Patients aged over 70.
6. Patients with chronic diseases. (***)
7. Patients with little social integration or limited help from relatives.

(*) Health literacy includes "all skills to independently obtain, understand and apply oral and written health information. This is independent of the understanding of the local language".
(**) Low literacy means "insufficient ability to read, spell or write in one's own mother tongue (illiteracy)".
(***) Chronic diseases: refers to health problems that require ongoing treatment over a period of years or decades. (World Health Organization, 2002)

Part 2: Patient pathways

We would like to get an idea of which wards patients with a potential COVID19 infection go through. The following questions relate to the appointment system, triage and referrals.

1. Indicate whether the statements are applicable to the appointment system of this practice:

- Yes
- No
- I do not know
- Does not apply

1. When patients want to make an appointment online at this practice, are they informed with which symptoms they are not allowed to enter the practice?
2. Do patients have to give a reason when making an appointment online at this practice?
3. Do patients have to give a reason when making an appointment by phone?

2. Indicate the extent to which you agree with the following statements regarding the appointment system in this practice:

- Never
- Rare

- Sometimes
- Mostly
- Always
- I do not know
- Does not apply

1. Patients who have made an appointment and for whom it is not clear whether they pose a risk of infection are called beforehand to check this.
2. In this practice, sufficient time is provided between consultations to disinfect the consulting room.
3. Home visits are organised so that potential COVID-19 patients are visited by the GP at the end of the home visit round.

3. Is there a consultation hour in this practice, which patients can visit without a prior appointment?
    - Yes
    - No
    - I do not know

4. To what extent does this practice use video consultations?
    - Never
    - Less than once a week
    - Weekly
    - Daily
    - Several times a day

<u>A. EVEN BEFORE THE COVID-19 PANDEMIC</u>
<u>B. SINCE THE START OF THE COVID-19 PANDEMIC</u>

5. Is a guide used for taking a call from potential COVID-19 patients?

Select one or more options:
- Yes, the guide is based on a state requirement
- Yes, the guide is not based on a state requirement
- No
- I do not know

6. How often is a detailed guide used when answering a call in this practice?
    - Never
    - Rare
    - Sometimes
    - Mostly
    - Always
    - I do not know
    - Does not apply

(This item is only relevant for practices that have a protocol as stated in question 4)

7. Does your telephone answering service ('the triaging person') receive assistance in assessing the call by a doctor/physician, if needed.

- Never
- Rare
- Sometimes
- Mostly
- Always
- I do not know
- Does not apply

8. Is the most up-to-date information regarding referral to a test centre available in each consultation room of this practice (e.g. procedure, telephone numbers, what documents need to be presented).

- Yes, this information is available in printed form
- Yes, this information is available in electronic form (e.g. on the computer desktop)
- No, the GP can find this information on a public website
- Other
- I do not know
- Does not apply

Explain 'Other': ________________________

9. Since the beginning of the pandemic, the role of non-physician staff may have changed. Please indicate to what extent you agree with the following statements.

- Do not agree at all
- Do not agree
- Neutral
- Agree
- Strongly agree
- I do not know/does not apply

1. Practice staff need to provide more information and recommendations to patients who contact the practice by phone.
2. Practice staff must more often explain what the GP has said to special patient groups (e.g. patients with a low level of education, with a migration background and/or low health literacy).
3. Practice staff are more involved in contacting patients who would otherwise postpone their health care themselves.
4. Practice staff are more involved in triage of patients (by telephone, on arrival in the practice,...).

10. Since the beginning of the pandemic, the role of doctors in this practice may have changed. Please indicate to what extent you agree with the following statements:

1. Doctors are more involved in contacting patients who would otherwise postpone their health care themselves.

- Do not agree at all

- Do not agree
- Neutral
- Agree
- Strongly agree
- I do not know
- Does not apply

11. Please indicate the extent to which you agree with the following statements regarding your role in the practice team in the COVID-19 pandemic:

- Do not agree at all
- Do not agree
- Neutral
- Agree
- Strongly agree
- I do not know
- Does not apply

1. My responsibility in this practice has increased.
2. I am satisfied with the change of tasks within my professional role.
3. I do not feel prepared for the change of tasks within my professional role.
4. I need further training for the changed tasks.

(This item is only to be completed by 'Other' as indicated in question 1)

12. Due to the complexity of GP care and the high level of uncertainty, unpredictable events may occur in GP practices. Please indicate whether the following events have occurred in this practice since the start of the COVID-19 pandemic:

- Yes
- No
- I do not know
- Does not apply

1. A patient with fever caused by a non-COVID infection was treated later due to following the practice's COVID 19 guide.
2. A patient with an acute illness was treated later because he/she did not come to the practice earlier.
3. A patient with a serious illness was treated later because he/she did not know how to contact the GP.
4. One patient with an acute illness was treated later because the situation was classified as non-acute by the telephone triage.
5. A patient with an acute illness that was not COVID-19 was misclassified in the triage process.

13. In this practice, one or more of the following initiatives have been taken since the beginning of the COVID-19 pandemic:

- Yes
- No

- I do not know

1. Electronic patient lists were created for patient groups with chronic diseases (e.g. patients taking methotrexate who need to be monitored).
2. This practice contacted patients with chronic diseases who need follow-up care.
3. This practice contacted psychiatrically vulnerable patients.
4. This practice contacted patients with previous domestic violence or child rearing issues.

(*) EPA = Electronic Patient Record

14. If a patient is referred to another facility (e.g. hospital, test centre), is it checked whether this patient is able to get there?
- Never
- Rare
- Sometimes
- Mostly
- Always
- I do not know

15. If a patient needs to isolate him/herself, the extent to which this is possible at home is checked.
- Never
- Rare
- Sometimes
- Mostly
- Always
- I do not know

16. Since the beginning of the COVID-19 pandemic, how often have patients talked to you about domestic violence?
- Not at all
- Less than before
- Just as much as before
- More than before
- Much more than before

17. How often did you check with patients to see if they had experienced domestic violence (in)directly since the onset of the COVID-19 pandemic?
- Not at all
- Less than before
- Just as much as before
- More than before
- Much more than before

(This item is only completed by GPs and doctors in training as indicated in question 1)

18. How often did you check whether patients (in-)directly experienced financial problems due to the COVID 19 pandemic?

- Not at all
- Less than before
- Just as much as before
- More than before
- Much more than before

(This item is only completed by GPs and doctors in training as indicated in question 1)

19. How often did patients talk to you about financial problems during the COVID-19 pandemic?

- Not at all
- Less than before
- Just as much as before
- More than before
- Much more than before

(This item is only completed by GPs and doctors in training as indicated in question 1)

Part 3: Infection prevention

Infection prevention is an important pillar when it comes to controlling infectious diseases. The following questions focus on different aspects of hygiene and disinfection protection as well as isolation policies in this practice.

1. Is the following equipment available in each consulting room in this practice?

- Yes
- No
- I do not know
- Does not apply

1. A washbasin
2. A water tap that can be operated with the elbow or by a motion detector
3. A rubbish bin that can be opened contactlessly
4. Disposable gloves
5. Disposable gown
6. Surface disinfectant
7. Paper to cover the examination table

2. In the following questions, we are interested in how the COVID-19 pandemic has changed infection prevention measures in this practice.

A. BEFORE THE COVID-19 PANDEMIC

- Always
- Sometimes

- Never

1A. At least one staff member wears nail polish.
2A. At least one staff member wears a ring or bracelet.
3A. For cleaning, the practice team uses a detailed hygiene plan (e.g. what is cleaned, frequency, method).
4A. Each consulting room is equipped with hand disinfectant. 5A. Hand sanitiser is provided for home visits.
6A. Hand disinfectant for patients is provided at the door or in the waiting room, for example.
7A. A separate home visit bag is provided for home visits to patients with suspected infection.

B. DURING THE COVID-19 PANDEMIC

- Always
- Sometimes
- Never

1A. At least one staff member wears nail polish.
2A. At least one staff member wears a ring or bracelet.
3A. For cleaning, the practice team uses a detailed hygiene plan (e.g. what is cleaned, frequency, method).
4A. Each consulting room is equipped with hand disinfectant. 5A. Hand sanitiser is provided for home visits.
6A. Hand disinfectant for patients is provided at the door or in the waiting room, for example.
7A. A separate home visit bag is provided for home visits to patients with suspected infection.

3. If COVID-19 suspected cases require other documents than prescriptions:

- Never
- Rare
- Sometimes
- Mostly
- Always
- I do not know
- Does not apply

1. ... are these documents available for collection from this practice?
2. ... are these documents sent to patients by post?
3. ... are these documents regularly sent to patients by e-mail?
4. ...these documents are available via an online system that complies with the General Data Protection Regulation (e.g. via a secure server to which patients have access - e.g. with a code).

4. The ambulatory care service is actively contacted by this practice when patients are diagnosed with a highly contagious disease.

- Never

- Rare
- Sometimes
- Mostly
- Always
- I do not know
-

1. ... patients who are diagnosed with COVID-19.
2. ... patients diagnosed with other dangerous and infectious diseases apart from COVID-10 (e.g.HIV, hepatitis carrier status).

Part 4: Information processing

In the following, we are interested in the extent to which the COVID-19 pandemic has changed how data and information are handled in this practice (e.g. policy updates and patient data).

1. In this practice, sufficient time is planned for GPs to be able to deal with new guidelines and relevant, reliable scientific literature.
    - Do not agree at all
    - Do not agree
    - Neutral
    - Agree
    - Strongly agree
    - I do not know

A. BEFORE THE COVID-19 PANDEMIC
B. SINCE THE START OF THE COVID-19 PANDEMIC

2. How often is a meeting called in this practice to discuss existing, new or changed guidelines?
    - Never
    - Less than once a week
    - Weekly
    - Daily
    - Several times a day
    - I do not know
    - Does not apply

A. BEFORE THE COVID-19 PANDEMIC
B. SINCE THE START OF THE COVID-19 PANDEMIC

Part 5: Patient information

1. How often was the information on this practice's website updated in the 12 months prior to the COVID-19 pandemic?
    - Not at all

- 1 or 2 times
- Less than once a month
- Once a month
- Weekly
- Daily
- I do not know
- This practice does not have a website

2. Is the patient information on this practice's website available in multiple languages?
   - Yes, in several languages
   - No
   - I do not know
   - This practice has no website

3. Is the information flyer of this practice available for patients in different languages?
   - Yes, in several languages
   - No
   - I do not know
   - This practice has no flyer

4. Does this practice have patient information on COVID-19?
   - Yes, in one language
   - Yes, in different languages
   - No
   - I do not know

5. Does the answering machine provide patient information in different languages?
   - Yes, in several languages
   - No
   - I do not know
   - There is no answering machine

Part 6: Cooperation, collegiality and self-protection

---

1. Within this practice team, is patient-related information that requires follow-up transferred? This can be both administrative and medical information.
   - Never
   - Rare
   - Sometimes
   - Mostly
   - Always
   - I do not know
   - Does not apply

2. If treatment errors occur in this practice, is this discussed in a team meeting (either with the practice team or only the doctors)?

- Never
- Rare
- Sometimes
- Mostly
- Always
- I do not know

3. Indicate to what extent you agree with the following statements:

- Do not agree at all
- Do not agree
- Neutral
- Agree
- Strongly agree
- I do not know

1. The government's targets during COVID-19 are endangering the practice organisation.
2. The targets set by the government during COVID-19 are endangering the health of the staff of this practice.
3. The government provides sufficient support for the smooth running of this practice.

4. Indicate to what extent you agree with the following statements:

- Do not agree at all
- Do not agree
- Neutral
- Agree
- Strongly agree
- I do not know

1. If members of the practice team are absent because of COVID-19 (due to infection or quarantine), can the work be distributed in such a way that the health of colleagues is not affected?
2. If members of the practice team are absent (due to infection or quarantine) because of COVID-19, can this practice count on the help of other practices in the area?
3. Has the COVID-19 pandemic encouraged cooperation with other practices in the area?

5. How does this practice ensure the well-being of the practice team during the COVID-19 pandemic? Select one or more answer options.

- Before patients are admitted to the practice, a triage is carried out
- The number of patients in the waiting room was reduced
- The waiting room is no longer used
- Infection control was increased
- Structural changes were made at the reception desk

- A triage is carried out by telephone
- Video consultations are carried out
- The handling of repeat prescriptions has been changed in such a way that patients no longer have to come to the practice for this.
- An electronic prescription is used or prescriptions are sent by post.

6. During the last month...
© Mayo Clinic Well-being Index
- Yes
- No

(This item is only answered by doctors, doctors in training, and 'Other' as in question 1 of part 1 indicated filled

1. Did you feel burnt out from work?
2. Have you been worried that your work is dulling you emotionally?
3. Did you feel down, depressed or hopeless?
4. Have you unintentionally fallen asleep in public?
5. Have tasks piled up so much that you can no longer manage them?
6. Have you been affected by emotional difficulties (such as anxiety, depression or irritability)?
7. Did you have physical impairments that no longer allowed you to manage your daily work at home and/or outside?

7. Please indicate to what extent you agree with the following statements:
© Mayo Clinic Well-being Index
(This item is only answered by doctors, doctors in training, and 'Other' as in question 1 of part 1 indicated filled in)

1 (Strongly disagree) 2 3 4 5 6 7 (Strongly agree)

1. The work I do has meaning for me.
2. The work I do has become more important since the COVID-19 pandemic began.

8. Please indicate to what extent you agree with the following statements:
© Mayo Clinic Well-being Index
(This item is only answered by doctors, doctors in further training, and 'other' as in question 1 of part 1 indicated filled in)

1 (Do not agree at all) 2 3 4 5 (Strongly agree)

1. My working hours leave me enough space for my private/family life.

9. Please indicate to what extent you agree with the following statements:
(This item is only answered by doctors, doctors in training, and 'other' as in question 1).
of part 1 indicated filled in)

1 (Strongly disagree) 2 3 4 5 6 (Strongly agree)

1. Despite the COVID-19 pandemic, my work leaves me enough space for my private life. /Family life.

10.How do you maintain your mental health? Write your answer in the box. ________________________________

Thank you for your participation. You have now completed all the questions in this questionnaire.

Lastly, we would like to thank you very much for your participation and would like to hear if you have any additional comments or remarks for us. Any feedback is welcome and may be written in the box. Don't forget to press 'Submit' to save your responses.

Write your answer in the box. _______________________
