# Supplementary material for: “The measures taken by the government overburdened the daily practice” – insights of the PRICOV-19 study on German general practitioners in times of COVID-19
Source: BMC Prim Care. 2023 Oct 11;24(Suppl 1):207. doi: 10.1186/s12875-023-02115-4 (PMC10568746; doi:10.1186/s12875-023-02115-4)
Supplement: Supplementary file 3 — Additional file 3. Main questionnaire, German. [file 12875_2023_2115_MOESM3_ESM.pdf]

Versorgungsqualität und Patientensicherheit in Hausarztpraxen zu Zeiten der Covid-19-Pandemie

Sehr geehrte Damen und Herren,

die COVID-19 Pandemie hat die hausärztliche Versorgung vor unvorhersehbare organisatorische und inhaltliche Herausforderungen gestellt, dazu gehören veränderte Aufgaben wie Telemedizin, die regionale Zusammenarbeit mit dem ÖGD, vertragsärztlichen und stationären Einrichtungen und begrenzte Verfügbarkeit personeller und infrastruktureller Ressourcen sowie fehlende personelle Schutzausrüstung (PSA). Das veränderte Arbeitsfeld stellt auch eine Herausforderung für die Qualität der Versorgung in ihren Dimensionen dar: Patientensicherheit, Effektivität, Patientenzentrierung, Aktualität, Effizienz und gesundheitliche Chancengleichheit.

Die PRICOV-19-Studie wird in mehr als 36 Ländern durchgeführt wird, um den Einfluss der COVID-19 Pandemie auf die Organisation der Versorgung in hausärztlichen Praxen einschätzen zu können. Zusätzlich wird untersucht, wie der Zugang zur Patientenversorgung sichergestellt wurde und welchen Einfluss die Pandemie auf die Versorgungsqualität nahm. Diese Studie wird international mit mehreren Kooperationspartnern aus verschiedenen Ländern durchgeführt, die Studienleitung obliegt der Universität Gent.

Ihre Teilnahme an dieser Studie bedeutet uns viel! Die Ergebnisse sollen die politischen Entscheidungsträger europaweit unterstützen, welche Maßnahmen die Versorgung im ambulanten Sektor in einer Pandemie verbessert werden.

Die Teilnahme an dieser Studie ist anonym und Ihre Antworten können nicht auf Sie zurückgeführt werden. Ihre Daten werden von der Universität Gent verwahrt und werden nur mit den kooperierenden ForscherInnen geteilt. Selbstverständlich werden Ihre Daten nach der europäischen Datenschutzgrundverordnung behandelt, nicht mit Dritten geteilt und nach 20 Jahren vernichtet.

Alle Forschungsergebnisse werden über die Websiten der beteiligten Forschungsgruppen, durch wissenschaftliche Veröffentlichungen und weitere Kommunikationskanäle öffentlich zugänglich gemacht.

Die Bearbeitung des Fragebogens wird circa 20 Minuten in Anspruch nehmen.
Wir bedanken uns im Voraus für Ihre Unterstützung!

Mit freundlichen Grüßen,

Prof. Dr. Thomas Kühlein
Stefanie Stark
Allgemeinmedizinisches Institut
Friedrich-Alexander-Universität Erlangen-Nürnberg

Prof. Dr. Sara Willems
Principal Investigator
Department of Public Health and Primary Care, Ghent University, Belgium

Ausfüllhilfe

Bitte beachten Sie folgende Hinweise zum Ausfüllen des Fragebogens:

- Arbeiten Sie in verschiedenen Praxen oder an mehreren Standorten? Dann füllen Sie den Fragebogen mit Bezug auf eine spezifische Praxis und Standort aus.
- Der Begriff ‚Mitarbeitende' im Fragebogen bezieht sich auf all jene, die aktiv in dieser Praxis arbeiten. Dies beinhaltet sowohl bezahlte als auch unbezahlte Mitarbeitende.
- Der Fragebogen sollte vorzugsweise durch einen Hausarzt/eine Hausärztin oder einen Arzt/eine Ärztin in Weiterbildung ausgefüllt werden. Sie können den Fragebogen außerdem als Team im Rahmen eines Teammeetings ausfüllen. In diesem Fall kann der Fragebogen sogar als Auslöser für teaminterne Diskussionen über die Qualitätssicherung in Zeiten von COVID-19 wirken.
- Es gibt keine falschen Antworten. Eventuell beinhalten die Antwortmöglichkeiten nicht die Antwort, die exakt Ihre Situation in der Praxis widerspiegelt. In diesem Falle wählen Sie bitte die Antwortmöglichkeit, die am ehesten der aktuellen Situation in Ihrer Praxis entspricht.
- Der Fragebogen besteht aus sechs Teilen: Hintergrundinformationen, Patientenpfade, Infektionsprävention, Informationsverarbeitung, Patienteninformation, und als Letztes Kooperation, Kollegialität und Selbstschutz.
- Es sollte nur ein Fragebogen pro Praxis ausgefüllt werden.

Das Ausfüllen des Fragebogens beträgt circa 20 Minuten.

Einwilligungserklärung

Ich habe die Informationen, die ich zu diese Studie erhalten habe, gelesen und verstanden.

- Ja
- Nein

Ich stimme zu, an dieser Studie teilzunehmen:

- Ja
- Nein

Teil 1: Hintergrundinformationen

Die folgenden Fragen beziehen sich auf Sie und die Strukturen dieser Praxis. Die darauffolgenden Fragen dienen der Einschätzung der aktuellen Situation in der Hausärztlichen Praxis (im Folgenden als ‚diese Praxis' bezeichnet).

1. Was ist Ihre Position in dieser Praxis?
   - Facharzt/Fachärztin für Allgemeinmedizin, praktische/r Arzt/Ärztin
   - Facharzt/Fachärztin für Innere Medizin (hausärztliche/r Internist/Internistin)
   - Arzt/Ärztin in Weiterbildung
   - Dieser Fragebogen wird als Praxisteam ausgefüllt
   - Sonstige

Erläutern Sie 'Sonstige': ________________

2. Wie viele Jahre Arbeitserfahrung haben Sie bereits im hausärztlichen Bereich nach Abschluss der Weiterbildung zum Facharzt für Allgemeinmedizin (*)?

- Jahr(e):_______________ (Schreiben Sie eine Zahl).
- Monat(e):_______________ (Schreiben Sie eine Zahl).

(*) Wenn Sie im hausärztlichen Bereich 1 Jahr und 2 Monate gearbeitet haben, füllen Sie bitte 1 Jahr(e) und 2 Monat(e) aus.

(Dieses Item wird nur von Ärzten/Ärztinnen, Arzt/Ärztin in Weiterbildung und 'Sonstige' wie in Frage 1 angegeben ausgefüllt)

3. Wie viele Personen arbeiten aktiv in dieser Praxis? (Sie eingeschlossen und unabhängig davon, ob bezahlt oder unbezahlt).

- Anzahl bezahlte Mitarbeitende (*) ________________ (Schreiben Sie eine Zahl).
- Anzahl unbezahlte Mitarbeitende (**):________________ (Schreiben Sie eine Zahl).

(*) Bezahlte Mitarbeitende: z.B. bezahlte Auszubildende, Verwaltungsangestellte, MFAs, Hausärzte/Hausärztinnen

(**) Unbezahlte Mitarbeitende: z.B. Studierende, PJ-ler, PraktikantInnen

4. Wie viele Hausärzte/Hausärztinnen und Arzt/Ärztinnen in Weiterbildung arbeiten in dieser Praxis? Zählen Sie jede/n Arzt/Ärztin und Arzt/Ärztin in Weiterbildung, unabhängig von ihrer Vollzeitäquivalenz (VZÄ). Vergessen Sie nicht, sich selbst mitzuzählen.

- Anzahl der Hausärzte/Hausärztinnen: ________________ (Schreiben Sie eine Zahl).
- Anzahl der Ärzte/Ärztinnen in Weiterbildung: ________________ (Schreiben Sie eine Zahl).

5. Wie viele Vollzeitäquivalenz-Hausärzte/Hausärztinnen (VZÄ) arbeiten in dieser Praxis(*)? Bitte geben Sie alle Hausärzte/Hausärztinnen an, inklusive der Ärzte/Ärztinnen in Weiterbildung und vergessen Sie nicht, sich selbst mitzuzählen. Bitte geben Sie die Zahl im Dezimalformat an (z.B. 2,5).

- Anzahl an VZÄs:________________ (Geben Sie eine Zahl an.)

(*) Bitte bedenken Sie, dass wir nach VZÄs fragen. Wenn also ein/e Hausärzt/Hausärztin drei Tage in der Woche arbeitet, so ist das ein VZÄ von 0,6.

6. Welche der folgenden Berufsgruppen sind in dieser Praxis vertreten? (Wundern Sie sich nicht, einige davon sind in anderen europäischen Ländern in Hausarztpraxen üblich)
(Geben Sie alle derzeit vertretenen Berufsgruppen an.)

- Hausarzt/Hausärztin
- Arzt/Ärztin in Weiterbildung
- Medizinische Fachangestellte
- Praxismananger/in
- Rezeptionist/in, Verwaltungsassistent/in
- Sozialarbeiter/in
- Reinigungskraft
- Podologe/in
- Psychologe/in
- Krankenschwester/pfleger oder Hilfsschwester/pfleger
- Physiotherapeut/in, manueller Therapeut/in, oder Osteopath/in
- Diätassistent/in oder Ernährungsberater/in
- Sonstige

Erläutern Sie 'Sonstige': ________________

7. Was ist das vorherrschende Honorarsystem(*) in dieser Praxis?

- Öffentliche Gebührenordnung (GOÄ)
  Einheitlicher Bewertungsmaßstab (EBM)
- Selektiv-Vertrag (n. §73b SGB V)
- Sonstige

Erläutern Sie 'Sonstige': ________________

(*) Vorherrschendes Honorarsystem: Das mit dem prozentual höchsten Anteil am Einkommen

8. Sind die Hausärzte/Hausärztinnen in dieser Praxis selbstständig oder in einem festen Angestelltenverhältnis? Bitte geben Sie alle Optionen an, die zutreffen.

- bezahltes Angestelltenverhältnis
- Selbstständig mit Vertrag bei einer Krankenkasse (in Deutschland mit Kassenzulassung)
- Selbstständig ohne Vertrag (reine Privatpraxis)

(Dieses Item wird nur von Ärzten/Ärztinnen wie in Frage 1 angegeben ausgefüllt)

9. Wie würden Sie die Lage Ihrer Praxis beschreiben?

- Großstadt (Innenstadt)
- Vorort
- (Klein-)Stadt
- Stadt-Land gemischt
- Ländlich

10. Haben Sie Einschränkungen bezüglich der Räumlichkeiten oder der Infrastruktur dieser Praxis wahrgenommen, die Sie seit Beginn der COVID-19 Pandemie daran hinderten, eine sichere und qualitativ hochwertige Versorgung zu gewährleisten?

- Zu großen Teilen
- In einigen Teilen
- Kaum
- Keine
- Keine Angabe

11. Hat die COVID-19 Pandemie bezüglich der Räumlichkeiten oder der Infrastruktur dieser Praxis dazu geführt, dass diesbezüglich Änderungen in Betracht gezogen werden?

- Zu großen Teilen
- In einigen Teilen
- Kaum
- Keine
- Keine Angabe

12. Wir würden gerne eine Vorstellung von der Größe dieser Praxis bekommen. Geben Sie eine Schätzung Ihrer gesamten Patientenpopulation an (NICHT-Scheinzahl pro Quartal).

- Anzahl Patienten: ________________ (Schreiben Sie eine Zahl.)

13. Verglichen mit der durchschnittlichen Hausarztpraxis, würden Sie sagen, dass diese Praxis im Durchschnitt mehr/weniger Patienten der unten gelisteten Kategorien behandelt?

- Über dem Durchschnitt
- Ungefähr durchschnittlich
- Unter dem Durchschnitt
- Ich weiß es nicht

1. Patienten mit Migrationshintergrund oder solche, die die Sprache vor Ort mit Mühe sprechen.
2. Patienten mit eingeschränkter Gesundheitskompetenz (*) oder niedriger Alphabetisierung (**).
3. Patienten mit finanziellen Problemen.
4. Patienten mit einer psychiatrischen Problemen.
5. Patienten im Alter über 70.
6. Patienten mit chronischen Krankheiten. (***)
7. Patienten mit geringer sozialer Einbindung oder eingeschränkter Hilfe durch Angehörige.

(*) Gesundheitskompetenz beinhaltet „alle Fähigkeiten, um mündliche und schriftliche Gesundheitsinformationen selbstständig zu beziehen, zu verstehen und anzuwenden. Dies ist unabhängig vom Verständnis der lokalen Sprache.“
(**) Niedrige Alphabetisierung bedeutet „ungenügende Fähigkeit zu lesen, zu buchstabieren oder zu schreiben in der eigenen Muttersprache (Analphabetisierung).”
(***) Chronische Krankheiten: bezieht sich auf gesundheitliche Probleme, die während einer Dauer von Jahren oder Jahrzehnten fortwährende Behandlung erfordern. (World Health Organization, 2002)

Teil 2: Patientenpfade

Wir würden gerne eine Vorstellung davon bekommen, welche Stationen Patienten und Patientinnen mit einer potentiellen COVID19-Infektion durchlaufen. Die folgenden Fragen beziehen sich auf das Terminsystem, Triage und Überweisungen.

1. Geben Sie an, ob die Aussagen auf das Terminsystem dieser Praxis anwendbar sind:

- Ja
- Nein
- Ich weiß es nicht
- Trifft nicht zu

1. Wenn Patienten online einen Termin in dieser Praxis vereinbaren wollen, werden sie darüber informiert, mit welchen Symptomen sie die Praxis nicht betreten dürfen?
2. Müssen Patienten einen Grund angeben, wenn sie online einen Termin in dieser Praxis vereinbaren?
3. Müssen Patienten müssen einen Grund angeben, wenn sie telefonisch einen Termin vereinbaren?

2. Geben Sie an, inwieweit Sie den folgenden Aussagen bezüglich des Terminsystems in dieser Praxis zustimmen:

- Nie
- Selten

- Manchmal
- Meistens
- Immer
- Ich weiß es nicht
- Trifft nicht zu

1. Patienten, die einen Termin vereinbart haben und bei denen es nicht eindeutig ist, ob sie eine Infektionsgefahr darstellen, werden vorher angerufen, um dies zu überprüfen.
2. In dieser Praxis wird ausreichend Zeit zwischen Konsultationen zur Verfügung gestellt, um das Sprechzimmer zu desinfizieren.
3. Die Hausbesuche werden so organisiert, dass potenzielle COVID-19 Patienten von dem Hausarzt/der Hausärztin am Ende der Hausbesuchrunde besucht werden.

3. Gibt es in dieser Praxis eine Sprechstunde, welche Patienten ohne vorherigen Termin aufsuchen können?
   - Ja
   - Nein
   - Ich weiß es nicht

4. Inwieweit nutzt diese Praxis Videokonsultationen?
   - Nie
   - Weniger als einmal die Woche
   - Wöchentlich
   - Täglich
   - Mehrere Male am Tag

<u>A. SCHON VOR DER COVID-19 PANDEMIE</u>
<u>B. SEIT BEGINN DER COVID-19 PANDEMIE</u>

5. Wird ein Leitfaden für das Entgegennehmen eines Anrufs von potentiellen COVID-19 Patienten benutzt?
Wählen Sie eine oder mehrere Optionen:
   - Ja, der Leitfaden basiert auf einer staatlichen Vorgabe
   - Ja, der Leitfaden basiert nicht auf einer staatlichen Vorgabe
   - Nein
   - Ich weiß es nicht

6. Wie oft wird beim Entgegennehmen eines Anrufs in dieser Praxis ein detaillierter Leitfaden verwendet?
   - Nie
   - Selten
   - Manchmal
   - Meistens
   - Immer
   - Ich weiß es nicht
   - Trifft nicht zu

(Dieses Item ist nur relevant für Praxen, die ein Protokoll haben wie in Frage 4 angegeben)

7. Erhält Ihre telefonische Annahme (‚die triagierende Person'), wenn benötigt, Unterstützung bei der Bewertung des Anrufs durch eine/n Arzt/Ärztin.

- Nie
- Selten
- Manchmal
- Meistens
- Immer
- Ich weiß es nicht
- Trifft nicht zu

8. Sind in jedem Sprechzimmer dieser Praxis die aktuellsten Informationen bezüglich der Überweisung an ein Testzentrum verfügbar (z.B. Vorgehen, Telefonnummern, welche Dokumente vorgelegt werden müssen).

- Ja, diese Informationen sind in gedruckter Form verfügbar
- Ja, diese Informationen sind in elektronischer Form verfügbar (z.B. auf dem Computer Desktop)
- Nein, der Hausarzt/die Hausärztin kann diese Informationen auf einer öffentlichen Website finden
- Sonstige
- Ich weiß es nicht
- Trifft nicht zu

Erläutern Sie 'Sonstige': ________________

9. Seit Beginn der Pandemie hat sich die Rolle der nicht-hausärztlichen Mitarbeitenden möglicherweise geändert. Bitte geben Sie an, inwieweit Sie den folgenden Aussagen zustimmen.

- Stimme überhaupt nicht zu
- Stimme nicht zu
- Neutral
- Stimme zu
- Stimme sehr zu
- Ich weiß es nicht/trifft nicht zu

1. Das Praxispersonal muss mehr Informationen und Empfehlungen an Patienten, die die Praxis telefonisch kontaktieren, weitergeben.
2. Das Praxispersonal muss häufiger, speziellen Patientengruppen (bspw. Patienten mit geringen Bildungsniveau, mit Migrationshintergrund und/oder geringer Gesundheitskompetenz), das was der Hausarzt/die Hausärztin gesagt hat, erklären.
3. Das Praxispersonal ist mehr daran beteiligt, Patienten zu kontaktieren, die sonst ihre Gesundheitsversorgung selbst aufschieben würden.
4. Das Praxispersonal ist mehr in die Triage der Patienten involviert (per Telefon, bei Ankunft in der Praxis,…).

10. Seit Beginn der Pandemie hat sich möglicherweise die Rolle der Ärzte/Ärztinnen in dieser Praxis geändert. Bitte geben Sie an, inwieweit Sie den folgenden Aussagen zustimmen:

1. Ärzte/Ärztinnen sind mehr daran beteiligt, Patienten zu kontaktieren, die sonst ihre Gesundheitsversorgung selbst aufschieben würden.

- Stimme überhaupt nicht zu

- Stimme nicht zu
- Neutral
- Stimme zu
- Stimme sehr zu
- Ich weiß es nicht
- Trifft nicht zu

11. Bitte geben Sie an, inwieweit Sie den folgenden Aussagen bezüglich Ihrer Rolle im Praxisteam in der COVID-19 Pandemie zustimmen:

- Stimme überhaupt nicht zu
- Stimme nicht zu
- Neutral
- Stimme zu
- Stimme sehr zu
- Ich weiß es nicht
- Trifft nicht zu

1. Meine Verantwortung in dieser Praxis ist gestiegen.
2. Mit der Veränderung der Aufgaben innerhalb meiner beruflichen Rolle bin ich zufrieden.
3. Ich fühle mich nicht vorbereitet auf die Veränderung der Aufgaben innerhalb meiner beruflichen Rolle.
4. Ich benötige eine Fortbildung für die veränderten Aufgaben.

(Dieses Item ist nur auszufüllen von 'Sonstige' wie in Frage 1 angegeben)

12. Aufgrund der Komplexität der hausärztlichen Versorgung und des hohen Maßes an Unsicherheit, können unvorhersehbare Ereignisse in den Hausarztpraxen vorkommen. Bitte geben Sie an, ob die folgenden Ereignisse in dieser Praxis seit Beginn der COVID-19 Pandemie aufgetreten sind:

- Ja
- Nein
- Ich weiß es nicht
- Trifft nicht zu

1. Ein/e Patient/in mit Fieber, ausgelöst durch eine Nicht-COVID-Infektion, wurde aufgrund des Befolgens des COVID-19-Leitfadens der Praxis, erst später behandelt.
2. Ein/e Patient/in mit einer akuten Krankheit wurde erst später behandelt, weil er/sie nicht früher in die Praxis kam.
3. Ein/e Patient/in mit einer ernsthaften Krankheit wurde erst später behandelt, weil er/sie nicht wusste, wie man den Hausarzt/die Hausärztin kontaktieren kann.
4. Ein/e Patient/in mit einer akuten Krankheit wurde später behandelt, weil die Situation durch die telefonische Triage als nicht akut eingestuft wurde.
5. Ein/e Patient/in mit einer akuten Krankheit, die nicht COVID-19 war, wurde im Triageprozesses falsch eingestuft.

13. In dieser Praxis wurden eine oder mehrere der folgenden Initiativen seit Beginn der COVID-19 Pandemie ergriffen:

- Ja
- Nein

- Ich weiß es nicht

1. Es wurden elektronische Patientenlisten für Patientengruppen mit chronischen Krankheiten ergestellt (z.B. Patienten/innen, die Methotrexat einnehmen und therapieüberwacht werden müssen).
2. Diese Praxis kontaktierte Patienten mit chronischen Krankheiten, die Nachversorgung benötigen.
3. Diese Praxis kontaktierte psychiatrisch gefährdete Patienten.
4. Diese Praxis kontaktierte Patienten mit früher aufgetretenen Problemen mit häuslicher Gewalt oder Problemen in der Kindeserziehung.

(*) EPA = Elektronische Patientenakte

14. Wenn ein/e Patient/in an eine andere Einrichtung (bspw. Krankenhaus, Testzentrum) überwiesen wird, wird überprüft, ob diese/r Patient/in in der Lage ist, dort hinzugelangen?
- Nie
- Selten
- Manchmal
- Meistens
- Immer
- Ich weiß es nicht

15. Wenn ein/e Patient/in sich isolieren muss, wird überprüft, inwieweit dies bei ihm/ihr zu Hause möglich ist.
- Nie
- Selten
- Manchmal
- Meistens
- Immer
- Ich weiß es nicht

16. Wie häufig haben Patienten seit Beginn der COVID-19 Pandemie mit Ihnen über häusliche Gewalt gesprochen?
- Überhaupt nicht
- Weniger als bisher
- Genauso viel wie bisher
- Mehr als bisher
- Viel mehr als bisher

17. Wie häufig haben Sie sich bei Patienten erkundigt, um festzustellen, ob sie (in)direkt seit Beginn der COVID-19 Pandemie häusliche Gewalt erfahren haben?
- Überhaupt nicht
- Weniger als bisher
- Genauso viel wie bisher
- Mehr als bisher
- Viel mehr als bisher

(Dieses Item wird nur von Hausärzten/Hausärztinnen und Ärzten/Ärztinnen in Weiterbildung wie in Frage 1 angegeben ausgefüllt)

18. Wie häufig haben Sie überprüft, ob Patienten (in-)direkt finanzielle Probleme aufgrund der COVID-19 Pandemie erfahren haben?

- Überhaupt nicht
- Weniger als bisher
- Genauso viel wie bisher
- Mehr als bisher
- Viel mehr als bisher

(Dieses Item wird nur von Hausärzten/Hausärztinnen und Ärzten/Ärztinnen in Weiterbildung wie in Frage 1 angegeben ausgefüllt)

19. Wie häufig haben Patienten mit Ihnen über finanzielle Probleme während der COVID-19 Pandemie gesprochen?

- Überhaupt nicht
- Weniger als bisher
- Genauso viel wie bisher
- Mehr als bisher
- Viel mehr als bisher

(Dieses Item wird nur von Hausärzten/Hausärztinnen und Ärzten/Ärztinnen in Weiterbildung wie in Frage 1 angegeben ausgefüllt)

Teil 3: Infektionsprävention

Infektionsprävention ist eine wichtige Säule, wenn es darum geht, infektiöse Krankheiten zu bekämpfen. Die folgenden Fragen konzentrieren sich auf verschiedene Aspekte des Hygiene- und Desinfektionsschutzes sowie der Isolationspolitik in dieser Praxis.

1. Ist in jedem Sprechzimmer in dieser Praxis folgende Ausstattung vorhanden?

- Ja
- Nein
- Ich weiß es nicht
- Trifft nicht zu

1. Ein Waschbecken
2. Ein Wasserhahn, der mit dem Ellenbogen oder durch einen Bewegungsmelder bedient werden kann
3. Ein Mülleimer, der kontaktlos geöffnet werden kann
4. Einweghandschuhe
5. Einwegkittel
6. Oberflächendesinfektionsmittel
7. Papier zum Abdecken des Untersuchungstisches

2. In den folgenden Fragen interessiert uns, inwiefern die COVID-19 Pandemie Maßnahmen zur Infektionsprävention in dieser Praxis verändert hat.

<u>A. VOR DER COVID-19 PANDEMIE</u>

- Immer
- Manchmal

- Nie

1A. Mindestens ein Mitarbeitender trägt Nagellack.
2A. Mindestens ein Mitarbeitender trägt einen Ring oder Armband.
3A. Zur Reinigung verwendet das Praxisteam einen detaillierten Hygiene-Plan (z.B. was gereinigt wird, Häufigkeit, Methode).
4A. Jedes Sprechzimmer ist mit Händedesinfektionsmittel ausgestattet.
5A. Händedesinfektionsmittel wird für Hausbesuche bereitgestellt.
6A. Händedesinfektionsmittel für Patienten wird beispielsweise an der Tür oder im Wartezimmer bereitgestellt.
7A. Für Hausbesuche bei Patienten mit mutmaßlicher Infektion wird eine separate Hausbesuchstasche bereitgestellt.

B. WÄHREND DER COVID-19 PANDEMIE

- Immer
- Manchmal
- Nie

1A. Mindestens ein Mitarbeitender trägt Nagellack.
2A. Mindestens ein Mitarbeitender trägt einen Ring oder Armband.
3A. Zur Reinigung verwendet das Praxisteam einen detaillierten Hygiene-Plan (z.B. was gereinigt wird, Häufigkeit, Methode).
4A. Jedes Sprechzimmer ist mit Händedesinfektionsmittel ausgestattet.
5A. Händedesinfektionsmittel wird für Hausbesuche bereitgestellt.
6A. Händedesinfektionsmittel für Patienten wird beispielsweise an der Tür oder im Wartezimmer bereitgestellt.
7A. Für Hausbesuche bei Patienten mit mutmaßlicher Infektion wird eine separate Hausbesuchstasche bereitgestellt.

3. Wenn COVID-19-Verdachtsfälle andere Dokumente außer Rezepte benötigen:

- Nie
- Selten
- Manchmal
- Meistens
- Immer
- Ich weiß es nicht
- Trifft nicht zu

1.… sind diese Dokumente zur Abholung in dieser Praxis verfügbar?
2.… werden diese Dokumente den Patienten postalisch zugestellt?
3.… werden diese Dokumente den Patienten regelmäßig per E-Mail zugeschickt?
4.…sind diese Dokumente über ein Onlinesystem verfügbar, das der allgemeinen Datenschutzverordnung entspricht (z.B. über einen sicheren Server, auf den die Patienten Zugriff haben - z.B. mit einem Kode).

4. Der ambulante Pflegedienst wird von dieser Praxis aktiv kontaktiert, wenn Patienten mit einer hoch ansteckenden Krankheit diagnostiziert werden.

- Nie

- Selten
- Manchmal
- Meistens
- Immer
- Ich weiß es nicht
-

1.… Patienten, die mit COVID-19 diagnostiziert werden.
2.… Patienten, die mit anderen gefährlichen und ansteckenden Krankheiten abseits von COVID-10 diagnostiziert werden (z.B.HIV, Hepatitis-Trägerstatus).

Teil 4: Informationsverarbeitung

Im Folgenden interessieren wir uns, inwieweit die COVID-19 Pandemie verändert hat, wie Daten und Informationen in dieser Praxis gehandhabt werden (z.B. Updates der Richtlinien und Patientendaten).

1. In dieser Praxis wird genügend Zeit für die Hausärzte/Hausärztinnen eingeplant, sich mit neuen Leitlininien und relevanter, verlässlicher wissenschaftlicher Literatur befassen zu können.
    - Stimme überhaupt nicht zu
    - Stimme nicht zu
    - Neutral
    - Stimme zu
    - Stimme sehr zu
    - Ich weiß es nicht

A. VOR DER COVID-19 PANDEMIE
B. SEIT BEGINN DER COVID-19 PANDEMIE

2. Wie oft wird in dieser Praxis eine Besprechung anberaumt, um existierende, neue oder geänderte Vorgaben zu diskutieren?
    - Nie
    - Weniger als einmal die Woche
    - Wöchentlich
    - Täglich
    - Mehrere Male am Tag
    - Ich weiß es nicht
    - Trifft nicht zu

A. VOR DER COVID-19 PANDEMIE
B. SEIT BEGINN DER COVID-19 PANDEMIE

Teil 5: Patienteninformation

1. Wie oft wurden die Informationen auf der Webseite dieser Praxis in den letzten 12 Monaten vor der COVID-19 Pandemie aktualisiert?
    - Überhaupt nicht

- 1 oder 2 Mal
- Weniger als einmal im Monat
- Einmal im Monat
- Wöchentlich
- Täglich
- Ich weiß es nicht
- Diese Praxis hat keine Webseite

2. Sind die Patienteninformation auf der Webseite dieser Praxis in mehreren Sprachen verfügbar?
   - Ja, in mehreren Sprachen
   - Nein
   - Ich weiß es nicht
   - Diese Praxis hat keine Webesite

3. Ist der Informationsflyer dieser Praxis für Patienten in verschiedenen Sprachen verfügbar?
   - Ja, in mehreren Sprachen
   - Nein
   - Ich weiß es nicht
   - Diese Praxis hat keinen Flyer

4. Hat diese Praxis eine Patienteninformationen zu COVID-19?
   - Ja, in einer Sprache
   - Ja, in verschiedenen Sprachen
   - Nein
   - Ich weiß es nicht

5. Stellt der Anrufbeantworter Patienteninformationen in verschiedenen Sprachen zur Verfügung?
   - Ja, in mehreren Sprachen
   - Nein
   - Ich weiß es nicht
   - Es gibt keinen Anrufbeantworter

Teil 6: Kooperation, Kollegialität und Selbstschutz

1. Werden innerhalb dieses Praxisteams, patientenbezogene Informationen, die eine Weiterverfolgung benötigen, übergeben? Dabei kann es sich sowohl um administrative als auch um medizinische Informationen handeln.
   - Nie
   - Selten
   - Manchmal
   - Meistens
   - Immer
   - Ich weiß es nicht
   - Trifft nicht zu

2. Wenn sich Behandlungsfehler in dieser Praxis ereignen, wird dies in einer Teambesprechung diskutiert (entweder mit dem Praxisteam oder nur den ÄrztInnen)?

- Nie
- Selten
- Manchmal
- Meistens
- Immer
- Ich weiß es nicht

3. Geben Sie an, inwieweit Sie den folgenden Aussagen zustimmen:

- Stimme überhaupt nicht zu
- Stimme nicht zu
- Neutral
- Stimme zu
- Stimme sehr zu
- Ich weiß es nicht

1. Die Vorgaben, der Regierung während der COVID-19 vorgibt, gefährden die Praxisorganisation.
2. Die Vorgaben, die die Regierung während der COVID-19 vorgibt, gefährden die Gesundheit der Mitarbeitende dieser Praxis.
3. Die Regierung stellt ausreichend Unterstützung für den reibungslosen Ablauf dieser Praxis zur Verfügung.

4. Geben Sie an, inwieweit Sie den folgenden Aussagen zustimmen:

- Stimme überhaupt nicht zu
- Stimme nicht zu
- Neutral
- Stimme zu
- Stimme sehr zu
- Ich weiß es nicht

1. Wenn Mitarbeitende des Praxisteams wegen COVID-19 ausfallen (wegen Infektion oder Quarantäne), kann die Arbeit so verteilt werden, dass die Gesundheit der Kollegen/Kolleginnen nicht beeinträchtig wird?
2. Wenn Mitarbeitende des Praxisteams wegen COVID-19 ausfallen (wegen Infektion oder Quarantäne) ausfallen, kann diese Praxis auf die Hilfe anderer Praxen in der Umgebung zählen?
3. Hat die COVID-19 Pandemie die Kooperation mit anderen Praxen in der Umgebung gefördert?

5. Wie stellt diese Praxis das Wohlergehen des Praxisteams während der COVID-19 Pandemie sicher? Wählen Sie eine oder mehrere Antwortmöglichkeiten aus.

- Vor Einlass der Patienten in die Praxis wird eine Triage durchgeführt
- Die Anzahl der Patienten im Wartezimmer wurde reduziert
- Das Wartezimmer wird nicht mehr verwendet
- Die Infektionskontrolle wurde erhöht
- Es wurden strukturelle Änderungen an der Rezeption vorgenommen

- Es wird telefonisch eine Triage durchgeführt
- Es werden Videokonsultationen durchgeführt
- Der Umgang mit Wiederholungsezepten wurde so verändert, dass Patienten/Patientinnen dafür nicht mehr in die Praxis kommen müssen
- Es wird ein elektronisches Rezept erwendet oder Rezepte per Post versendet

6. Während des letzten Monats...
© Mayo Clinic Well-being Index
  - Ja
  - Nein

(Dieses Item wird nur von Ärzten/Ärztinnen, Arzt/Ärztin in Weiterbildung, und 'Sonstige' wie in Frage 1 von Teil 1 angegeben ausgefüllt

1. Fühlten Sie Sich von der Arbeit ausgebrannt?
2. Haben Sie Sich Sorgen gemacht, dass Ihre Arbeit Sie emotional abstumpft?
3. Fühlten Sie Sich niedergeschlagen, depressiv oder hoffnungslos?
4. Sind Sie ungewollt in der Öffentlichkeit eingeschlafen?
5. Haben sich Aufgaben so angesammelt, dass Sie diese nicht mehr bewältigen können?
6. Wurden Sie durch emotionale Schwierigkeiten (wie Ängstlichkeit, Depression oder Reizbarkeit) beeinträchtigt?
7. Hatten Sie physische Beeinträchtigungen, die die Bewältigung Ihrer alltäglichen Arbeit zuhause und/oder außerhalb nicht mehr ermöglichten?

7. Bitte geben Sie an, inwieweit Sie den folgenden Aussagen zustimmen:
© Mayo Clinic Well-being Index
(Dieses Item wird nur von Ärzten/Ärztinnen, Arzt/Ärztin in Weiterbildung, und 'Sonstige' wie in Frage 1 von Teil 1 angegeben ausgefüllt)

1 (Stimme überhaupt nicht zu) 2 3 4 5 6 7 (Stimme sehr zu)

1. Die Arbeit, die ich mache, hat eine Bedeutung für mich.
2. Die Arbeit, die ich mache, hat seit Beginn der COVID-19 Pandemie an Bedeutung gewonnen.

8. Bitte geben Sie an, inwieweit Sie den folgenden Aussagen zustimmen:
© Mayo Clinic Well-being Index
(Dieses Item wird nur von Ärzten/Ärztinnen, Arzt/Ärztin in Weiterbildung, und 'Sonstige' wie in Frage 1 von Teil 1 angegeben ausgefüllt)

1 (Stimme überhaupt nicht zu) 2 3 4 5 (Stimme sehr zu)

1. Meine Arbeitszeiten lassen mir genug Raum für mein Privat-/Familienleben.

9. Bitte geben Sie an, inwieweit Sie den folgenden Aussagen zustimmen:
  - ➔ *(Dieses Item wird nur von Ärzten/Ärztinnen, Arzt/Ärztin in Weiterbildung, und 'Sonstige' wie in Frage 1 von Teil 1 angegeben ausgefüllt)*

1 (Stimme überhaupt nicht zu) 2 3 4 5 6 (Stimme sehr zu)

1. Trotz der COVID-19 Pandemie, lässt mir meine Arbeit genug Raum für mein Privat- /Familienleben.

10.Wie erhalten Sie Ihre geistige Gesundheit aufrecht?
Schreiben Sie Ihre Antwort in die Box. ________________

Vielen Dank für Ihre Teilnahme. Sie haben nun alle Fragen dieses Fragebogens ausgefüllt.

Als Letztes möchten wir uns für Ihre Teilnahme herzlich bedanken und würden gerne erfahren, ob Sie zusätzlichen Kommentare oder Anmerkungen für uns haben. Jegliches Feedback ist willkommen und darf in die Box geschrieben werden. Vergessen Sie nicht 'Absenden' zu drücken, um Ihre Antworten zu speichern.

Schreiben Sie Ihre Antwort in die Box. ________________
