# Supplementary material for: “The measures taken by the government overburdened the daily practice” – insights of the PRICOV-19 study on German general practitioners in times of COVID-19
Source: BMC Prim Care. 2023 Oct 11;24(Suppl 1):207. doi: 10.1186/s12875-023-02115-4 (PMC10568746; doi:10.1186/s12875-023-02115-4)
Supplement: Supplementary file 4 — Additional file 4. Items added, English. [file 12875_2023_2115_MOESM4_ESM.pdf]

1. Indicate to what extent you agree with the following statements:

- Do not agree at all
- Do not agree
- Neutral
- Agree
- Strongly agree
- I do not know

a. COVID-19 suspected cases are given priority when appointments are made.
b. The care of COVID (suspected) cases cannot sufficiently ensure the care of uncomplicated diseases (e.g. back pain, urinary tract infection).
c. Patients with uncomplicated diseases (e.g. back pain, urinary tract infection) are currently mainly treated by telephone consultations.
d. Suspected COVID-19 cases are mainly treated by means of telephone consultations.
e. No appointments are currently offered for routine examinations (e.g. health check-ups, disease management programmes).

2. Indicate to what extent you agree with the following statements:

- Do not agree at all
- Do not agree
- Neutral
- Agree
- Strongly agree
- I do not know

a. The role of GPs has gained attention in society since the beginning of the pandemic.
b. GP care has become more important for ensuring general patient care since the beginning of the pandemic.
c. Since the beginning of the pandemic, the focus of GP activity has shifted towards the increased documentation effort (e.g. certificates, communication with the public health department, changed billing).
d. The measures taken by the government regarding GP care to contain the pandemic have overwhelmed everyday practice.

e. Sufficient support has been provided by the government to be able to meet the specified measures.

3. **Indicate to what extent you agree with the following statements:**

- Do not agree at all
- Do not agree
- Neutral
- Agree
- Strongly agree
- I do not know

a. The possibility of a telephone or video consultation relieves the burden on practice resources.
b. Since the beginning of the pandemic, our practice has increasingly offered home visits to patients at risk (e.g. with multimorbidity).
c. The local structures of medical cooperation (e.g. interprofessional exchange, substitute organisation) have changed positively as a result of the pandemic.
d. Protective vaccinations (e.g. influenza, pneumococci) are increasingly recommended by our practice on the grounds of the pandemic.
e. The request for COVID-19 testing by asymptomatic patients is increasing.
f. COVID-19 testing of asymptomatic patients is increasing as the pandemic progresses.
