# Supplementary material for: “The measures taken by the government overburdened the daily practice” – insights of the PRICOV-19 study on German general practitioners in times of COVID-19
Source: BMC Prim Care. 2023 Oct 11;24(Suppl 1):207. doi: 10.1186/s12875-023-02115-4 (PMC10568746; doi:10.1186/s12875-023-02115-4)
Supplement: Supplementary file 5 — Additional file 5. Items added, German. [file 12875_2023_2115_MOESM5_ESM.pdf]

1. Geben Sie an, inwieweit Sie den folgenden Aussagen zustimmen:

- Stimme überhaupt nicht zu
- Stimme nicht zu
- Neutral
- Stimme zu
- Stimme sehr zu
- Ich weiß es nicht

a. COVID-19-Verdachtsfälle werden bei der Terminvergabe bevorzugt behandelt.
b. Durch die Versorgung der COVID-(Verdachts)Fälle kann die Versorgung von unkomplizierten Erkrankungen (z.B. Rückenschmerzen, Harnwegsinfekt) nicht ausreichend sichergestellt werden.
c. PatientInnen mit unkomplizierten Erkrankungen (z.B. Rückenschmerzen, Harnwegsinfekt) werden aktuell überwiegend mittels telefonischer Sprechstunde versorgt.
d. COVID-19-Verdachtsfälle werden überwiegend mittels telefonischer Sprechstunde versorgt.
e. Für Routineuntersuchungen (z.B. Gesundheitsuntersuchungen, Disease Management Programme) werden aktuell keine Termine angeboten.

2. Geben Sie an, inwieweit Sie den folgenden Aussagen zustimmen:

- Stimme überhaupt nicht zu
- Stimme nicht zu
- Neutral
- Stimme zu
- Stimme sehr zu
- Ich weiß es nicht

a. Die Rolle der Hausärzteschaft hat seit Beginn der Pandemie in der Gesellschaft an Aufmerksamkeit gewonnen.
b. Die hausärztliche Versorgung hat seit Beginn der Pandemie für die Sicherstellung der allgemeinen Patientenversorgung an Bedeutung gewonnen.
c. Der Schwerpunkt der hausärztlichen Tätigkeit hat sich seit Beginn der Pandemie in Richtung des vermehrten Dokumentationsaufwandes (z.B. Atteste, Kommunikation mit dem Gesundheitsamt, veränderte Abrechnungen) verschoben.
d. Die Maßnahmen, die seitens der Regierung bezüglich der hausärztlichen Versorgung zur Eindämmung der Pandemie getroffen worden sind, haben den Praxisalltag überfordert.

e. Seitens der Regierung wurden ausreichen Unterstützungsangebote bereitgestellt, um den vorgegebenen Maßnahmen gerecht werden zu können.

3. Geben Sie an, inwieweit Sie den folgenden Aussagen zustimmen:

- Stimme überhaupt nicht zu
- Stimme nicht zu
- Neutral
- Stimme zu
- Stimme sehr zu
- Ich weiß es nicht

a. Die Möglichkeit einer Telefon- oder Videosprechstunde entlastet die Praxisressourcen.
b. In unserer Praxis werden seit Beginn der Pandemie bei Risikopatienten (z.B. mit Multimorbidität) vermehrt Hausbesuche angeboten.
c. Die lokalen Strukturen der ärztlichen Zusammenarbeit (z.B. interprofessioneller Austausch, Vertretungsorganisation) haben sich durch die Pandemie positiv verändert.
d. Schutzimpfungen (z.B. Influenza, Pneumokokken) werden von unserer Praxis mit Begründung der Pandemie vermehrt empfohlen.
e. Die Anfrage nach COVID-19-Testungen durch asymptomatische Patienten nimmt zu.
f. Die Durchführung von COVID-19-Testungen bei asymptomatischen Patienten nimmt im Verlauf der Pandemie zu.
