# Supplementary material for: “The measures taken by the government overburdened the daily practice” – insights of the PRICOV-19 study on German general practitioners in times of COVID-19
Source: BMC Prim Care. 2023 Oct 11;24(Suppl 1):207. doi: 10.1186/s12875-023-02115-4 (PMC10568746; doi:10.1186/s12875-023-02115-4)
Supplement: Supplementary file 7 — Additional file 7. Syntax. [file 12875_2023_2115_MOESM7_ESM.pdf]

```
* Encoding: UTF-8.
* Encoding: .

**************************
***PRICOV-19***
**************************

Get FILE=
*********************************************************************************

**Fragebogenbezogene Daten:

*Anzahl Bögen:

FREQUENCIES Variables=my_first_instrument_complete
 /STATISTICS=STDDEV MINIMUM MAXIMUM MEAN MEDIAN SUM SKEWNESS SESKEW
  /ORDER=ANALYSIS.

*komplett ausgefüllt: 250 Bögen (71,6%), unkomplett: 99 Bögen (28,4%), gesamt: 349 Bögen
(100,0%)

*Wie viele Bögen wurden wann ausgefüllt, Angabe in Kategorie: Tage; Feldphase: 01.02. bis
28.02.2021:

FREQUENCIES Variables=week_survey
 /STATISTICS=STDDEV MINIMUM MAXIMUM MEAN MEDIAN SUM SKEWNESS SESKEW
  /ORDER=ANALYSIS.

*Zeit des Ausfüllens/Rücklaufs bei insgesamt 250 Bögen im Februar erfasst; bei 99 '(28,4) Bögen nicht
erfasst:
01.02. bis 07.02. = 82 (23,5) Bögen
08.02. bis  14.02. = 54 (15,5) Bögen
15.02. bis 21.02. = 78 (22,3) Bögen
22.02. bis 28.02. = 36 (10,3) Bögen


FREQUENCIES feedback.

*Feedback-Anzahl (offenes Antwortfeld, FraBo Ende): 83 Feedbacks


***Praxisbezogene Daten:

*Art/Position der Teilnehmenden in der Praxis, die Bogen ausgefüllt haben:

FREQUENCIES VARIABLES=participant_function
  /STATISTICS=STDDEV MINIMUM MAXIMUM MEAN MEDIAN SUM SKEWNESS SESKEW
  /ORDER=ANALYSIS.

*Mittelwert der Befragen: 208 GPs*
```

```
Compute position=participant_function.
Recode position (0=1) (1=2) (2=3) (3=3) (4=3) (5=3).
FREQUENCIES position
  /STATISTICS=STDDEV MINIMUM MAXIMUM MEAN MEDIAN SUM SKEWNESS SESKEW
  /ORDER=ANALYSIS.

*1= HA, 2=ÄiW, 3= hausärztlich tätiger Internist*


*Dummy position, für Regression:

COMPUTE GP = position.
IF position = 1 GP = 1.
IF position <> 1 GP = 0.

Compute Trainee = position.
IF position = 2 Trainee = 1.
IF position <> 2 Trainee = 0.

Compute Internist = position.
IF position =3 Internist = 1.
IF position<> 3 Internist = 0.

FREQUENCIES GP.
FREQUENCIES Trainee.
FREQUENCIES Internist.


*Berufserfahrung; kategorial:

FREQUENCIES VARIABLES=participant_experience_cat_5yrs
  /STATISTICS=STDDEV MINIMUM MAXIMUM MEAN MEDIAN SUM SKEWNESS SESKEW
  /ORDER=ANALYSIS.

*Dummy-Bildung für Regression & Splitt-off

*Mittelwert: Befragte haben zwischen 15-20 Jahre Berufserfahrung, metrische kategorial Vriable,
kann so verwendet werden.

Compute less_workexperience = participant_experience_cat_5yrs.
    If participant_experience_cat_5yrs <=2 less_workexperience = 1.
If participant_experience_cat_5yrs > 2 less_workexperience = 0.

*weniger als 15 Jahre Berufserfahrung (Median=15).

Compute lot_workexperience = participant_experience_cat_5yrs.
    If participant_experience_cat_5yrs >2 lot_workexperience = 1.
If participant_experience_cat_5yrs <= 2 lot_workexperience = 0.

*Mehr als 15 Jahre Berufserfahrung.
```

```
FREQUENCIES VARIABLES=less_workexperience lot_workexperience
  /STATISTICS=STDDEV MINIMUM MAXIMUM MEAN MEDIAN SUM SKEWNESS SESKEW
  /ORDER=ANALYSIS.

*ab 15 Jahre Berufserfahrung = höhere Berufserfahrung.

*Lokale Struktur der Praxis, gruppiert:

FREQUENCIES VARIABLES=pcp_location
  /STATISTICS=STDDEV MINIMUM MAXIMUM MEAN MEDIAN SUM SKEWNESS SESKEW
  /ORDER=ANALYSIS.

*Großstadt: 43 (12,3)
Vorstadt: 33 (9,5)
Kleinstadt: 56 (16,0)
Stadt-Land gemischt: 39 (11,2)
Land: 91 (26,1)
Gesamt: 262 (75,1); keine Antwort: 87 (24,9)
Land und Kleinstadt am Häufigsten vertreten.

*Dummy-Bildung, für Regression & Splitt-off:

*Stadt:

Compute urban = pcp_location.
If pcp_location <= 2 urban = 1.
If pcp_location >2 urban = 0.
Frequencies urban.


*Land:

Compute rural = pcp_location.
If pcp_location > 2 rural = 1.
If pcp_location <= 2 rural = 0.
FREQUENCIES rural.


*Praxisgröße anhand Patientenanzahl:

FREQUENCIES VARIABLES=pcp_patients
  /STATISTICS=STDDEV MINIMUM MAXIMUM MEAN MEDIAN SUM SKEWNESS SESKEW
  /ORDER=ANALYSIS.

Compute practice_size=pcp_patients.
FREQUENCIES VARIABLES=practice_size
    /STATISTICS=STDDEV MINIMUM MAXIMUM MEAN MEDIAN SUM SKEWNESS SESKEW
  /ORDER=ANALYSIS.

*Mittelwert: 5375, Median:3000.
```

```
*Einzel- oder Gemeinschaftspraxis*

*Einzelpraxis*

Compute Single_sum = pcp_gp_statute___3 + pcp_gp_statute___4.
FREQUENCIES VARIABLES= single_sum.

*Dummy, für Regression :

Compute Single_practice = Single_sum.
IF Single_sum = 0 Single_practice = 0.
IF Single_sum = 1 Single_practice = 1.
IF Single_sum = 2 Single_practice = 1.
FREQUENCIES Single_practice
  /STATISTICS=STDDEV MINIMUM MAXIMUM MEAN MEDIAN SUM SKEWNESS SESKEW
  /ORDER=ANALYSIS.

*220 Einzelpraxen

*Gemeinschaftspraxis*

FREQUENCIES pcp_gp_statute___1.
*Die Variable hat nur fehlende Werte, trifft also nie zu und kann ignoriert werden.

FREQUENCIES pcp_gp_statute___2.
COMPUTE Joint_practice = pcp_gp_statute___2.
FREQUENCIES Joint_practice.

FREQUENCIES VARIABLES=Joint_practice
  /STATISTICS=STDDEV MINIMUM MAXIMUM MEAN MEDIAN SUM SKEWNESS SESKEW
  /ORDER=ANALYSIS.

*111 Gemeinschaftspraxen


*Cohort characteristics Dummies gesamt, als Übersicht für Regressionen:

FREQUENCIES Variables = GP Trainee Internist urban rural less_workexperience lot_workexperience
practice_size Single_practice Joint_practice
  /STATISTICS=STDDEV MINIMUM MAXIMUM MEAN MEDIAN SUM SKEWNESS SESKEW
  /ORDER=ANALYSIS.


*******************************************************************************
************************************

***Deskriptive Analyse: Häufigkeiten, Verteilungen etc.***

***Cohort characteristics, ohne dummies, detailliert:
```

FREQUENCIES Variables=position participant_experience_cat_5yrs pcp_location practice_size Single_practice Joint_practice
    /STATISTICS=STDDEV MINIMUM MAXIMUM MEAN MEDIAN SUM SKEWNESS SESKEW
  /ORDER=ANALYSIS.

*Wahrnehmung der deutschen HÄ bezüglich der Pandemieauswirkungen in der Praxis; deutsche Fragen.
Diese Ergebnisse werden nur deskriptiv dargestellt, um aufzuzeigen, wie die deutschen HÄ die Pandemie wahrgenommen haben. Dient zur Bekräftigung der Hypothese.

FREQUENCIES VARIABLES = De_ptflow_appt_1
De_ptflow_appt_2
De_ptflow_appt_3
De_ptflow_appt_4
De_ptflow_appt_5
De_collab_org_1
De_collab_org_2
De_collab_org_3
De_collab_org_4
De_collab_org_5
De_collab_org_6
 /STATISTICS=STDDEV MINIMUM MAXIMUM MEAN MEDIAN SUM
  /ORDER=ANALYSIS.

*Split off nach location, Berufserfahrung, Einzel- oder Gemeinschaftspraxis, um zu wissen wer was wie beantwortet hat, da Characteristics wie es aussieht in Regression keinen Einfluss haben:
 Split offs stummgeschalten, auch hier kamen keine interessanten Unterschiede bei raus...
*Sort Cases by urban rural.
*Split File by urban rural.
  *   Frequencies De_ptflow_appt_1
De_ptflow_appt_2
De_ptflow_appt_3
De_ptflow_appt_4
De_ptflow_appt_5
De_collab_org_1
De_collab_org_2
De_collab_org_3
De_collab_org_4
De_collab_org_5
De_collab_org_6
        /statistics=all.
*Split File off.

*Kaum Unterschiede in Median & Mean vorhanden.

*Sort Cases by less_workexperience lot_workexperience.
*Split File by less_workexperience lot_workexperience.
  *   Frequencies De_ptflow_appt_1
De_ptflow_appt_2

De_ptflow_appt_3
De_ptflow_appt_4
De_ptflow_appt_5
De_collab_org_1
De_collab_org_2
De_collab_org_3
De_collab_org_4
De_collab_org_5
De_collab_org_6
/statistics=all.
*Split File off.

*Kaum Unterschiede in der Beantwortung vorhanden.

*Sort Cases by Joint_practice Single_practice.
*Split File by Joint_practice Single_practice.
* Frequencies De_ptflow_appt_1
De_ptflow_appt_2
De_ptflow_appt_3
De_ptflow_appt_4
De_ptflow_appt_5
De_collab_org_1
De_collab_org_2
De_collab_org_3
De_collab_org_4
De_collab_org_5
De_collab_org_6
/statistics=all.
*Split File off.

*Unterschiede der Beantwortung ob in einer Einzel oder Gemeinschaftspraxis sind vorhanden.

***Wahrnehmung der Veränderung der Rolle und Aufgaben der HÄ::

FREQUENCIES Variables=De_collab_society_1
De_collab_society_2
De_collab_society_3
participant_role_1
participant_role_2
participant_role_3
/STATISTICS=STDDEV MINIMUM MAXIMUM MEAN MEDIAN SUM
/ORDER=ANALYSIS.

*Splitt off nach location, Berufserfahrung, Einzel- oder Gemeinschaftspraxis, um zu wissen wer was wie beantwortet hat, da Characteristics in Regression keinen Einfluss haben:

*Sort Cases by urban rural.
*Split File by urban rural.
* Frequencies De_collab_society_1
De_collab_society_2

```
De_collab_society_3
participant_role_1
participant_role_2
participant_role_3
        /statistics=all.
*Split File off.

*Sort Cases by less_workexperience lot_workexperience.
*Split File by less_workexperience lot_workexperience.
*Frequencies De_collab_society_1
De_collab_society_2
De_collab_society_3
participant_role_1
participant_role_2
participant_role_3
        /statistics=all.
*Split File off.

*Sort Cases by Joint_practice Single_practice.
*Split File by Joint_practice Single_practice.
*Frequencies De_collab_society_1
De_collab_society_2
De_collab_society_3
participant_role_1
participant_role_2
participant_role_3
        /statistics=all.
*Split File off.

*Dummy-Bildung, für Regression, da als einzelne AVs verwendet:

Compute role_gained_attention = De_collab_society_1.
IF De_collab_society_1 <= 2 role_gained_attention = 0.
IF De_collab_society_1 = 3 role_gained_attention = 1.
IF De_collab_society_1 >3 role_gained_attention = 1.
FREQUENCIES role_gained_attention.

Compute phc_more_important = De_collab_society_2.
IF De_collab_society_2 <= 2 phc_more_important = 0.
IF De_collab_society_2 = 3 phc_more_important = 1.
IF De_collab_society_2 >3 phc_more_important = 1.
FREQUENCIES phc_more_important.


Compute phc_changed = De_collab_society_3.
IF De_collab_society_3 <= 2 phc_changed = 0.
IF De_collab_society_3 = 3 phc_changed = 1.
IF De_collab_society_3 >3 phc_changed = 1.
FREQUENCIES phc_changed.

Compute responsibilites_increased = participant_role_1.
```

```
IF participant_role_1 <= 1 responsibilites_increased = 0.
IF participant_role_1 = 2 responsibilites_increased = 1.
IF participant_role_1 >2 responsibilites_increased = 1.
FREQUENCIES responsibilites_increased.

Compute happy_taskshifting = participant_role_2.
IF participant_role_2 <= 1 happy_taskshifting = 0.
IF participant_role_2 = 2 happy_taskshifting = 1.
IF participant_role_2 >2 happy_taskshifting = 1.
FREQUENCIES happy_taskshifting.


Compute not_feel_prepared = participant_role_3.
IF participant_role_3 <= 1 not_feel_prepared = 0.
IF participant_role_3 = 2 not_feel_prepared = 1.
IF participant_role_3 >2 not_feel_prepared = 1.
FREQUENCIES not_feel_prepared.


FREQUENCIES VARIABLES = role_gained_attention phc_more_important phc_changed
responsibilites_increased happy_taskshifting not_feel_prepared
    /STATISTICS=STDDEV MINIMUM MAXIMUM MEAN MEDIAN SUM
  /ORDER=ANALYSIS.


***Well-being der HÄ (expanded 9-item Mayo Clinic Wellbeing Index):

FREQUENCIES Variables=participant_mcwi_emotionalexhaustion
participant_mcwi_depersonalisation
participant_mcwi_depression
participant_mcwi_fatigue
participant_mcwi_stress
participant_mcwi_mentalqol
participant_mcwi_physicalqol
participant_mcwi_meaning
participant_mcwi_schedule
 /STATISTICS=STDDEV MINIMUM MAXIMUM MEAN MEDIAN SUM
  /ORDER=ANALYSIS.


*Sort Cases by urban rural.
*Split File by urban rural.
 *     Frequencies participant_mcwi_emotionalexhaustion
participant_mcwi_depersonalisation
participant_mcwi_depression
participant_mcwi_fatigue
participant_mcwi_stress
participant_mcwi_mentalqol
participant_mcwi_physicalqol
participant_mcwi_meaning
participant_mcwi_schedule
```

```
        /statistics=all.
*Split File off.

*Sort Cases by less_workexperience lot_workexperience.
*Split File by less_workexperience lot_workexperience.
*Frequencies participant_mcwi_emotionalexhaustion
participant_mcwi_depersonalisation
participant_mcwi_depression
participant_mcwi_fatigue
participant_mcwi_stress
participant_mcwi_mentalqol
participant_mcwi_physicalqol
participant_mcwi_meaning
participant_mcwi_schedule
        /statistics=all.
*Split File off.

*Sort Cases by Joint_practice Single_practice.
*Split File by Joint_practice Single_practice.
*Frequencies participant_mcwi_emotionalexhaustion
participant_mcwi_depersonalisation
participant_mcwi_depression
participant_mcwi_fatigue
participant_mcwi_stress
participant_mcwi_mentalqol
participant_mcwi_physicalqol
participant_mcwi_meaning
participant_mcwi_schedule
        /statistics=all.
*Split File off.


*Dummybildung für Index, nach Vorgabe des Mayo Clinic Wellbeing Index*

*Meaningful, 7-Likert*

COMPUTE meaningfull_dummy = participant_mcwi_meaning.
IF participant_mcwi_meaning = 1 meaningfull_dummy = 1.
IF participant_mcwi_meaning = 2 meaningfull_dummy = 1.
IF participant_mcwi_meaning = 3 meaningfull_dummy = 0.
IF participant_mcwi_meaning = 4 meaningfull_dummy = 0.
IF participant_mcwi_meaning = 5 meaningfull_dummy = 0.
IF participant_mcwi_meaning = 6 meaningfull_dummy = -1.
IF participant_mcwi_meaning = 7 meaningfull_dummy = -1.

FREQUENCIES meaningfull_dummy
/STATISTICS=STDDEV MINIMUM MAXIMUM MEAN MEDIAN SUM
/ORDER=ANALYSIS.

*sieht gut aus*
```

```
*Work-life-balance, 5-Likert *

COMPUTE worklife_dummy = participant_mcwi_schedule.
IF participant_mcwi_schedule = 1 worklife_dummy = 1.
IF participant_mcwi_schedule = 2 worklife_dummy = 1.
IF participant_mcwi_schedule = 3 worklife_dummy = 0.
IF participant_mcwi_schedule = 4 worklife_dummy = -1.
IF participant_mcwi_schedule = 5 worklife_dummy = -1.

FREQUENCIES worklife_dummy
/STATISTICS=STDDEV MINIMUM MAXIMUM MEAN MEDIAN SUM
/ORDER=ANALYSIS.

*sieht gut aus*



***Bewertung der geundheitspolitischen Maßnahmen:

FREQUENCIES Collab_government_1 Collab_government_2 Collab_government_3
De_collab_society_4
 /STATISTICS=STDDEV MINIMUM MAXIMUM MEAN MEDIAN SUM
 /ORDER=ANALYSIS.

*Splitt off nach location, Berufserfahrung, Einzel- oder Gemeinschaftspraxis, um zu wissen wer was
wie beantwortet hat, da Characteristics in Regression keinen Einfluss haben:

*Sort Cases by urban rural.
*Split File by urban rural.
  *    Frequencies Collab_government_1 Collab_government_2 Collab_government_3
De_collab_society_4
        /statistics=all.
*Split File off.

*Sort Cases by less_workexperience lot_workexperience.
*Split File by less_workexperience lot_workexperience.
*Frequencies Collab_government_1 Collab_government_2 Collab_government_3
De_collab_society_4
        /statistics=all.
*Split File off.

*Sort Cases by Joint_practice Single_practice.
*Split File by Joint_practice Single_practice.
*Frequencies Collab_government_1 Collab_government_2 Collab_government_3
De_collab_society_4
        /statistics=all.
*Split File off.

*Dummybildung um Index für Regression bilden zu können: -1, 0, +1:

Compute threat_to_organisation = Collab_government_1.
IF Collab_government_1 <= 1 threat_to_organisation = -1.
```

```
IF Collab_government_1 = 2 threat_to_organisation = 0.
IF Collab_government_1 >2 threat_to_organisation = 1.
FREQUENCIES threat_to_organisation.

Compute threat_to_wellbeing = Collab_government_2.
IF Collab_government_2 <= 1 threat_to_wellbeing = -1.
IF Collab_government_2 = 2 threat_to_wellbeing = 0.
IF Collab_government_2 >2 threat_to_wellbeing = 1.
FREQUENCIES threat_to_wellbeing.

Compute inadequate_support = Collab_government_3.
IF Collab_government_3 <= 1 inadequate_support = 1.
IF Collab_government_3 = 2 inadequate_support = 0.
IF Collab_government_3 >2 inadequate_support = -1.
FREQUENCIES inadequate_support.

Compute policy_overloaded_practice = De_collab_society_4.
IF De_collab_society_4 <= 2  policy_overloaded_practice = -1.
IF De_collab_society_4 = 3  policy_overloaded_practice = 0.
IF De_collab_society_4 >3  policy_overloaded_practice= 1.
FREQUENCIES  policy_overloaded_practice.


FREQUENCIES Variables=threat_to_organisation  threat_to_wellbeing inadequate_support
policy_overloaded_practice
 /STATISTICS=STDDEV MINIMUM MAXIMUM MEAN MEDIAN SUM
  /ORDER=ANALYSIS.



*********************************************************************************
*****************

****Indexbildung für Regressionen um als UVs nutzen zu können****

*WellbeingIndex:

*Cronbachs alphha checken, ob Variablen für additiven Index geeignet (Kontrolle, da in previous
studies bereits erprobtes Isntrument):

RELIABILITY
  /VARIABLES=participant_mcwi_emotionalexhaustion
 participant_mcwi_depersonalisation
 participant_mcwi_depression
 participant_mcwi_fatigue
participant_mcwi_stress
participant_mcwi_mentalqol
participant_mcwi_physicalqol
meaningfull_dummy
worklife_dummy
  /SCALE('ALL VARIABLES') ALL
```

```
  /MODEL=ALPHA
  /STATISTICS=DESCRIPTIVE CORR
  /SUMMARY=TOTAL.

*Passt: Cronbach über 0.7*

*Additiver Index aus allen Variablen:

COMPUTE WellbeingIndex = participant_mcwi_emotionalexhaustion +
    participant_mcwi_depersonalisation + participant_mcwi_depression + participant_mcwi_fatigue +
participant_mcwi_stress + participant_mcwi_mentalqol + participant_mcwi_physicalqol +
meaningfull_dummy + worklife_dummy.

FREQUENCIES VARIABLES=WellbeingIndex
  /STATISTICS=STDDEV MEAN MEDIAN MINIMUM MAXIMUM
  /HISTOGRAM
  /ORDER=ANALYSIS.

**********************************************************************************
**************************************
*PolicyIndex:

*Cronbachs alphha checken, ob Variablen für Index geeignet:

RELIABILITY
  /VARIABLES=threat_to_organisation  threat_to_wellbeing inadequate_support
policy_overloaded_practice
  /SCALE('ALL VARIABLES') ALL
  /MODEL=ALPHA
  /STATISTICS=DESCRIPTIVE CORR
  /SUMMARY=TOTAL.

*Sieht gut aus, Cronbachs alpha 0.692, Index kann gebildet werden.

*Additiver Index aus allen 4 Variablen.
COMPUTE PolicyIndex = threat_to_organisation + threat_to_wellbeing + inadequate_support +
policy_overloaded_practice.
FREQUENCIES VARIABLES=PolicyIndex
  /STATISTICS=STDDEV MEAN MEDIAN MINIMUM MAXIMUM
  /HISTOGRAM
  /ORDER=ANALYSIS.

**********************************************************************************
************
*Zur Übersicht und besseren Darstellung der Tendenzen.
*Dummy Erstellung um ggf. überischtlich deskriptiv auswerten zu können & für spätere
Interpretation*


*WellbeingIndex zu Dummy, Trennung bei Median=1.0000:
```

```
COMPUTE WellbeingIndex_dummy = WellbeingIndex.
IF WellbeingIndex < 1 WellbeingIndex_dummy = 0.
IF WellbeingIndex >= 1 WellbeingIndex_dummy = 1.

FREQUENCIES WellbeingIndex_dummy
/STATISTICS=STDDEV MINIMUM MAXIMUM MEAN MEDIAN SUM
/ORDER=ANALYSIS.

******
*PolicyIndex zu Dummy, Trennung bei Median=1.0000:

COMPUTE PolicyIndex_dummy = PolicyIndex.
IF PolicyIndex < 1 PolicyIndex_dummy = 0.
IF PolicyIndex >= 1 PolicyIndex_dummy = 1.

FREQUENCIES PolicyIndex_dummy
/STATISTICS=STDDEV MINIMUM MAXIMUM MEAN MEDIAN SUM
/ORDER=ANALYSIS.


************************************************************************************
***********

***Regressionsanalysen***

***Regressionsmodelle:
*Die Modelle führen die UVs in drei Stufen ein, zunächst die Praxis-relevanten Variablen, dann die
Rollen- und Aufgabenwahrnehmungs Variablen, dann die jeweiligen Indexe*


************************************************************************************
*************

*Regression AV PolicyIndex*

*Wie wirkt sich das Wellbeing (die mentale Geundheit) auf die politische Einstellung der GP aus,
ohne weitere Modelle.

REGRESSION
 /MISSING LISTWISE
/STATISTICS COEFF OUTS R ANOVA COLLIN TOL
 /CRITERIA=PIN(.05) POUT(.10)
/NOORIGIN
/DEPENDENT PolicyIndex
/METHOD=ENTER WellbeingIndex
/SCATTERPLOT=(*ZRESID ,*ZPRED)
/RESIDUALS DURBIN HISTOGRAM(ZRESID)
/CASEWISE PLOT(ZRESID) OUTLIERS(3).

*Signifikant!
```

****

*3 stufiges Mixed Models Regression

*Nehmen Wellbeing (Index) + die Veränderungen der Rolle und Aufgaben der HÄ , Einfluss auf die Wahrnehmung und Einstellung der GPs bzgl. der gesundheitspolitischen Maßnahmen (Index):

```
REGRESSION
/MISSING LISTWISE
 /STATISTICS COEFF OUTS R ANOVA COLLIN TOL
/CRITERIA=PIN(.05) POUT(.10)
/NOORIGIN
/DEPENDENT PolicyIndex
/METHOD=ENTER phc_changed responsibilites_increased happy_taskshifting not_feel_prepared
/METHOD=ENTER WellbeingIndex
/METHOD=ENTER GP urban rural Single_practice less_workexperience
/SCATTERPLOT=(*ZRESID ,*ZPRED)
/SCATTERPLOT=(*ZRESID ,*ZPRED)
/RESIDUALS DURBIN HISTOGRAM(ZRESID)
/CASEWISE PLOT(ZRESID) OUTLIERS(3).
```

Einige signifikante Ergebnisse, passt! Hypothes kann bestätigt werden! ;-)

****
****

****
****
****
KONTROLL-REGRESSIONfließt nicht in Auswertung mit ein**umgedrehte Regression bestätigt erstes Modell!*

Regression AV WellbeingIndex

*Wie wirkt sich die politische Einstellung der GP auf das eigene Wohlbefinden.

```
REGRESSION
 /MISSING LISTWISE
/STATISTICS COEFF OUTS R ANOVA COLLIN TOL
 /CRITERIA=PIN(.05) POUT(.10)
/NOORIGIN
/DEPENDENT WellbeingIndex
/METHOD=ENTER PolicyIndex
/SCATTERPLOT=(*ZRESID ,*ZPRED)
/RESIDUALS DURBIN HISTOGRAM(ZRESID)
/CASEWISE PLOT(ZRESID) OUTLIERS(3).
```

*Signifikant!
****
KONTROLLE

*Nehmen die Veränderungen der Rolle und Aufgaben der HÄ sowie die Wahrnehmung und Einstellung der GPs bzgl. der gesundheitspolitischen Maßnahmen, Einfluss auf das wellbeing der HÄ:

```
REGRESSION
 /MISSING LISTWISE
/STATISTICS COEFF OUTS R ANOVA COLLIN TOL
 /CRITERIA=PIN(.05) POUT(.10)
/NOORIGIN
/DEPENDENT WellbeingIndex
/METHOD=ENTER phc_changed responsibilites_increased happy_taskshifting not_feel_prepared
/METHOD=ENTER PolicyIndex
/METHOD=ENTER GP urban rural Single_practice less_workexperience
/SCATTERPLOT=(*ZRESID ,*ZPRED)
/RESIDUALS DURBIN HISTOGRAM(ZRESID)
/CASEWISE PLOT(ZRESID) OUTLIERS(3).
*********************************************************************************
*************************************************************
*****************************
   ***Ende!***
```
